# CRISPR-mediated *Bmpr2* point mutation exacerbates late pulmonary vasculopathy and reduces survival in rats with experimental pulmonary hypertension

**DOI:** 10.1186/s12931-022-02005-w

**Published:** 2022-04-08

**Authors:** Jane Chanda Kabwe, Hirofumi Sawada, Yoshihide Mitani, Hironori Oshita, Naoki Tsuboya, Erquan Zhang, Junko Maruyama, Yoshiki Miyasaka, Hideyoshi Ko, Kazunobu Oya, Hiromasa Ito, Noriko Yodoya, Shoichiro Otsuki, Hiroyuki Ohashi, Ryuji Okamoto, Kaoru Dohi, Yuhei Nishimura, Tomoji Mashimo, Masahiro Hirayama, Kazuo Maruyama

**Affiliations:** 1 The Department of Anesthesiology and Critical Care Medicine, Mie University Graduate School of Medicine, 2-174 Edobashi, Tsu city, Mie 514-8507 Japan; 2 The Department of Pediatrics, Mie University Graduate School of Medicine, Mie, Japan; 3 The Department of Clinical Engineering, Suzuka University of Medical Science, Mie, Japan; 4 Institute of Experimental Animal Sciences, Osaka University Graduate School of Medicine, Osaka, Japan; 5 The Department of Cardiology and Nephrology, Mie University Graduate School of Medicine, Mie, Japan; 6 The Department of Integrative Pharmacology, Mie University Graduate School of Medicine, Mie, Japan; 7 Laboratory Animal Research Center, The Institute of Medical Science, The University of Tokyo, Tokyo, Japan; 8 The Department of Pediatrics, Nagoya City University School of Medicine, Aichi, Japan; 9 The Department of Neonatology, Fuzhou Children’s Hospital of Fujian Province, Fujian Medical University, Fujian, China

**Keywords:** Pulmonary hypertension, Survival, Rats, Genome editing, Animal model

## Abstract

**Background:**

Patients with pulmonary arterial hypertension (PAH) carrying bone morphogenetic protein receptor type 2 (*Bmpr2*) mutations present earlier with severe hemodynamic compromise and have poorer survival outcomes than those without mutation. The mechanism underlying the worsening clinical phenotype of PAH with *Bmpr2* mutations has been largely unaddressed in rat models of pulmonary hypertension (PH) because of the difficulty in reproducing progressive PH in mice and genetic modification in rats. We tested whether a clinically-relevant *Bmpr2* mutation affects the progressive features of monocrotaline (MCT) induced-PH in rats.

**Methods:**

A monoallelic single nucleotide insertion in exon 1 of *Bmpr2* (+/44insG) was generated in rats using clustered regularly interspaced short palindromic repeats (CRISPR)/CRISPR-associated protein 9, then PH, pulmonary vascular disease (PVD) and survival after MCT injection with or without a phosphodiesterase type 5 inhibitor, tadalafil, administration were assessed.

**Results:**

The +/44insG rats had reduced BMPR2 signalling in the lungs compared with wild-type. PH and PVD assessed at 3-weeks after MCT injection were similar in wild-type and +/44insG rats. However, survival at 4-weeks after MCT injection was significantly reduced in +/44insG rats. Among the rats surviving at 4-weeks after MCT administration, +/44insG rats had increased weight ratio of right ventricle to left ventricle plus septum (RV/[LV + S]) and % medial wall thickness (MWT) in pulmonary arteries (PAs). Immunohistochemical analysis showed increased vessels with Ki67-positive cells in the lungs, decreased mature and increased immature smooth muscle cell phenotype markers in the PAs in +/44insG rats compared with wild-type at 3-weeks after MCT injection. Contraction of PA in response to prostaglandin-F2α and endothelin-1 were significantly reduced in the +/44insG rats. The +/44insG rats that had received tadalafil had a worse survival with a significant increase in RV/(LV + S), %MWT in distal PAs and RV myocardial fibrosis compared with wild-type.

**Conclusions:**

The present study demonstrates that the *Bmpr2* mutation promotes dedifferentiation of PA smooth muscle cells, late PVD and RV myocardial fibrosis and adversely impacts both the natural and post-treatment courses of MCT-PH in rats with significant effects only in the late stages and warrants preclinical studies using this new genetic model to optimize treatment outcomes of heritable PAH.

**Supplementary Information:**

The online version contains supplementary material available at 10.1186/s12931-022-02005-w.

## Background

Pulmonary arterial hypertension (PAH) is a serious and progressive disease characterized by increased pulmonary vascular resistance due to occlusive pulmonary vascular disease (PVD), leading to right heart failure and ultimately death [1, 2]. Since 70% of heritable and 15–40% of apparently sporadic PAH develop on the basis of monoallelic bone morphogenetic protein type 2 receptor (*Bmpr2*) mutations, *Bmpr2* is recognized as the major genetic risk factor for developing PAH [3]. Clinically, PAH patients with *Bmpr2* mutations have been shown to have early onset, poor prognosis, reduced vascular reactivity, impaired right ventricular function, and resistance to PAH-specific therapy [2, 4–7]. Experimentally, haploinsufficiency of *Bmpr2* has been implicated in the development of PVD and PAH pathology by impeding pulmonary endothelial and smooth muscle cell functions including apoptosis, proliferation, differentiation and inflammation and right ventricular function [2, 8, 9]. The role of *Bmpr2* has also been extensively studied in models of pulmonary hypertension (PH) using genetically modified mice [10]. However, it is difficult to assess the role of *Bmpr2* in the development of progressive PVD and its relevance to survival, because of the difficulty in recapitulating the progressive features of human PAH in mouse models [11]. Thus, the role of *Bmpr2* mutations in the clinical phenotype, particularly the difference in survival between mutation carrier and non-carrier and its association with the development of PVD, has rarely been explored in experimental PH models.

In rats, administration of monocrotaline (MCT) induces progressive and fatal PH [12] (MCT-PH) and exposure to chronic hypoxia following SU5416 injection reproduces lung pathology characteristic of human PAH, including neointima formation and plexiform lesions [13]. In MCT-PH in rats, a single administration of MCT results in early vascular endothelial damage, followed by muscularization of the distal pulmonary artery (PA) and then an increase in pulmonary arterial pressure (PAP) is observed 2 weeks after MCT injection [11, 14]. PAP further increases along with progression of PVD and reduction of BMPR2 expression in the lung [15, 16] and rats appear to start dying 3-weeks after MCT injection [11, 12]. These features of MCT-PH allow us to replicate the onset and outcome of clinical PAH in rats. Thus, the recent introduction of genome editing techniques could be useful for PH research due to their ability to induce mutations into larger laboratory animals, including rats [17]. In fact, using rat models with monoallelic *Bmpr2* mutation in which 16 to 527 base pairs of the *Bmpr2* gene were deleted by zinc finger nuclease (ZFN), showed spontaneous PH with low genetic penetrance [18] and inflammatory stimulus-induced PH [19].

In the present study, we created a rat model in which a single nucleotide insertion was introduced into *Bmpr2* using efficient and newer method of clustered regularly interspaced short palindromic repeats (CRISPR) and CRISPR-associated protein 9 (Cas9) genome editing, and tested whether the monoallelic *Bmpr2* mutation affects MCT-induced progressive PH in rats.

## Methods

This article has supplemental tables and figures which are available in the Additional file 1.

### Ethics statement

Animal care, the experimental procedures, protocols for all animal experiments were approved by the Animal Research Ethics Committee, Mie University School of Medicine (Number 2019-28). All animal experiments were carried out in accordance with the National Institute of Health Guide for the Care and Use of Laboratory Animals (NIH Publication). Catheterization and tissue harvest were performed under anesthesia using intraperitoneal sodium pentobarbital. Echocardiography was performed under anesthesia using isoflurane. During the course of the experiment, ethical endpoints were indicated based on feeding and watering difficulties, agonizing symptoms (e.g., dyspnea, self-injurious behaviour), prolonged external abnormalities (e.g., diarrhoea, bleeding) with no signs of recovery, and rapid weight loss (> 20% in a few days). All efforts were made to minimize pain perception in the animal studies.

### Generation of rats with CRISPR/Cas9-induced *Bmpr2* mutation

**Fig. 1:**
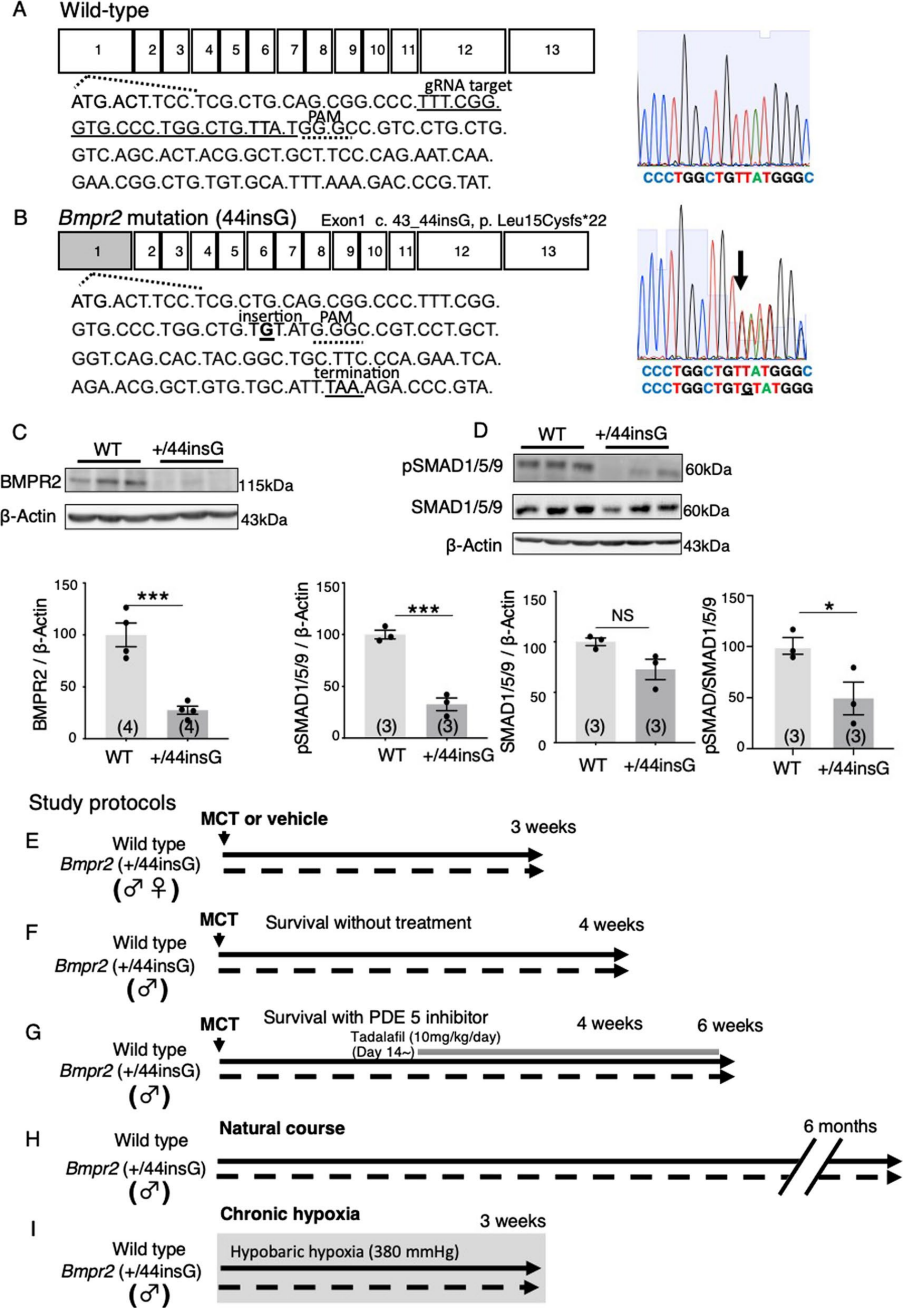
Generation of rats with *Bmpr2* mutation using CRISPR/Cas9. **A** Wild-type (WT) *Bmpr2* gene. Guide RNA (gRNA) was designed to target the sequence indicated by underline next to protospacer adjacent motif (PAM, GGG indicated by dashed underline). (Top right) The electropherograms with single waveforms in WT rat. **B** A single nucleotide (guanine) insertion at position 43–44 (G in bold and underline) in the exon 1 of BMPR2 coding DNA (designated as “44insG”), causes a frameshift at amino acid 15 (Leu15Cys) and results into *Bmpr2* translation termination (TAA indicated by underline) at amino acid 37 (22 amino acid down from the first frameshift). (Bottom right) The electropherograms with double waveforms after the site of mutation in a +/44insG rat. **C**, **D** Dysregulated BMPR2 signalling in the lung of the +/44insG rats at 7 weeks of age. Representative Western blots and the quantification of relative expression of BMPR2 protein (**C**, n = 4 for each group) and its downstream substrate proteins SMAD1/5/9 (n = 3 for each group), phosphorylated SMAD1/5/9 (n = 3 for each group) and the pSMAD/SMAD1/5/9 ratio (**D**). β-actin was used as the loading control. **E**–**I** Schematic illustration of the study protocols. Seven-week-old rats were injected with monocrotaline (MCT, 60 mg/kg) or saline as indicated. **E **Assessment at 3 weeks after MCT or saline injection in both male and female +/44insG and WT rats (n = 43 for male and n = 23 for females). **F** Survival study in male rats, 4 weeks following MCT injection in both +/44insG and WT littermates without treatment (n = 32). **G** Survival with a phosphodiesterase type 5 inhibitor, tadalafil (10 mg/kg) therapy from day 14 to day 28 (n = 18) and from day 14 to day 42 (n = 27) after MCT injection in male +/44insG and WT littermates. **H** Natural course of male +/44insG and WT littermates at 6 months of age. **I** Chronic hypoxia model for male +/44insG and WT littermates (n = 11). Evaluation was done after 3 weeks of exposure to hypobaric hypoxia (380 mmHg). Data are presented as means ± SEM; unpaired t-test; *p < 0.05, ***p < 0.001. Numbers 1–13 = Exon numbers; gRNA = guide RNA; PAM = protospacer adjacent motif; A = adenine; G = guanine; C = cytosine; T = thymine; BMPR2 = bone morphogenetic protein receptor type 2; WT = wild-type; +/44insG = *Bmpr2* mutant rat; MCT = monocrotaline;  = female rats  =  male rats

Clustered regularly interspaced short palindromic repeats (CRISPR) and CRISPR-associated protein 9 (Cas9) genome editing was used to generate rats with a *Bmpr2* mutation (Fig. 1A and B) [20]. The technique for the animal knockout system by electroporation (TAKE) method and CRISPR/Cas9 system was performed to generate the *Bmpr2* heterozygous mutant rats as previously described [21].

Cas9 mRNA was transcribed in vitro using an mMESSAGE mMACHINE T7 Ultra Kit (Life Technologies, Carlsbad, CA, USA) from a linearized plasmid (ID #72602; www.addgene.org/CRISPR) and was purified using a MEGAClear kit (Life Technologies). Specific crRNAs were purchased from Integrated DNA Technologies inc. (IDT, Coralville, IA) (Alt-R CRISPR-Cas9 crRNA) and were assembled with a tracrRNA (Alt-R CRISPR-Cas9 tracrRNA) before use according to the manufacturer's instructions. The fertilized eggs collected from Jcl:SD female rats (CLEA Japan, Inc. Tokyo, Japan) were used for the generation of the founder rats. For electroporation (EP), pronuclear embryos were placed in 40 µl of serum-free media (Opti-MEM Thermo Fisher Scientific, MA, USA) containing 400 ng/µl Cas9 mRNA and 200 ng/µl gRNA. In groups of 50–100, embryos were deposited into a 5 mm gap electrode (CUY505P5 or CUY520P5, Nepa Gene, Chiba, Japan) and electroporated using a NEPA21 Super Electroporator (Nepa Gene). The EP's poring pulses were voltage 225 V, pulse width 2.0 ms, pulse interval 50 ms, and a number of pulses + 4. The first and second transfer pulses were voltage 20 V, pulse width 50 ms, pulse interval 50 ms, and a number of pulses + 5. Embryos that developed to the two-cell stage after introducing RNAs were transferred into the oviducts of female surrogates anesthetized with isoflurane (DS Pharma Animal Health Co., Ltd., Osaka, Japan).

These rats, which were generated in the manner described above, were subsequently mated with Sprague–Dawley rats (Japan SLC, Inc. Shizuoka, Japan) to have first generation (F1) offspring with either heterozygous *Bmpr2* mutation or wild-type. Confirmation of the genetic makeup was done by direct sequencing of genomic DNA. At four weeks of age, the rats were sedated with isoflurane, and approximately 2 mm^3^ of distal rat tail was cut under aseptic technique for extraction of DNA using Kapa Express Extract Buffer (10X) KB7101 (KapaBiosystems, Wilmington, MA) and Kapa Express Extract Enzyme KE7102 (KapaBiosystems). The tail and mix were put in the thermocycler machine (PCR GeneAmp® PCR 2700, Applied Biosystems, Foster City, CA) for digestion at 75ºC for 10 min followed by enzyme inactivation at 95 ℃ for 5 min. DNA was recovered and subjected to PCR with Kapa2G Robust HotStart ReadyMix (2X) KM5702 (KapaBiosystems), by using the primers GAAACTACACGTAACACCGAAGGT (r-*BMPR2*-R 82244753 M9493 (AO3), Thermo Fisher Scientific) and ATCTGCCATTTGTCCTTTCAAACTG (r-*BMPR2*-F 82244753 M9493 (AO2), Thermo Fisher Scientific). The PCR was performed in thermocycler as follows: initial 95 ºC/5 min, which was followed by 30 cycles of (94 ºC/1 min; 98 ºC/10 s; 60 ºC/15 s and 68 ºC/30 s). The PCR products were treated with ExoSAP-IT® (Thermo Fisher Scientific P/N 78201 Lot: 4335327) before conducting the sequencing. Direct sequencing of the PCR products was performed with a 3130xl Genetic Analyzer (Applied Biosystems). The genome sequences of the F1 rats were confirmed by sanger sequencing (FASMAC, Kanagawa, Japan). Thereafter, the next and subsequent generations of rats were obtained by sibling mating and used in experiments.

### Animal model

The seven-week-old male or female offspring with either mutant or wild-type (WT) were used for the experiment assessed at 3 weeks after MCT and only male rats were used for the rest of experiments, kept under standard laboratory conditions and fed a laboratory diet and water ad libitum. To establish experimental PH, rats weighing 200-300 g were injected subcutaneously with monocrotaline (MCT, 60 mg/kg body weight, Sigma Aldrich, St. Louis, MO) or saline and assessed pulmonary hemodynamics, pulmonary vascular disease (PVD) and survival at the different time points depending on the study group. To establish chronic hypoxia induced PH, rats were exposed to hypobaric hypoxia (380 mmHg, equivalent to 10% O_2_) for 3 weeks as previously described [22].The rats with heterozygous *Bmpr2* mutation and wild-type littermates, designated as +/44insG and WT respectively, were assigned to the following study groups (Fig. 1E–I): assessment at 3 weeks after MCT injection (n = 43 [male], 23[female]); survival without treatment at day 28 MCT (n = 32, male only); survival with a phosphodiesterase (PDE) type 5 inhibitor, tadalafil, treatment (n = 18 [treatment to day 28], n = 27 [treatment to day 42], male only); evaluation at 6 months of age (n = 15, male only); evaluation after 3 weeks of chronic hypoxic exposure (n = 11, male only). For the PDE type 5 inhibitor treatment study, 10 mg/kg body weight of tadalafil (Sigma Aldrich) were given orally daily from day 14 up to day 28, while another group received same dose of tadalafil only up to day 42 after MCT injection. Tadalafil is an approved oral phosphodiesterase type-5 inhibitor used in clinical practice which inhibits the breakdown of cyclic guanosine monophosphate (cGMP) thereby inducing pulmonary vasorelaxation.

### Hemodynamic studies

At the designated time of analysis, the rats were anaesthetized with intraperitoneal sodium pentobarbital (30 mg/kg) to perform the hemodynamic studies. A Silascon tubing (0.31 mm inner [ID] and 0.64 mm outer [OD] diameters respectively) were inserted into the right pulmonary artery (PA) via the right external jugular vein initially and then the right ventricle by closed-chest technique [22, 23]. The parameters assessed were mean pulmonary arterial pressure (mPAP), right ventricular systolic pressure (RVSP). Another short Silascon tubing about 10 mm (0.30 mm ID and 0.60 mm OD, respectively) serving as the tip was connected to a polyethylene tubing (0.35 mm ID × 1.05 mmOD, KN-392-SP19, Natsume Seisakusho Co., Ltd, Tokyo, Japan) and this was inserted into the right internal carotid artery to measure the systemic arterial pressure. Blood was also collected to assess hematocrit.

### Echocardiography

For echocardiography, the rats were anesthetized using 3% isoflurane in 3 L/min of oxygen and maintained at 1.5–2% isoflurane in 2 L/min of oxygen. Transthoracic echocardiography was performed using a Vevo 2100 imaging system (FUJIFILM VisualSonics Inc.) with the high-frequency (13–24 MHz) MS-250 transducer. Transthoracic two-dimensional and pulsed-wave Doppler modes were used to obtain the triplet of the main pulmonary artery (PA) diameter measurements and velocity–time integral measurements. Echocardiography-derived cardiac output, calculated using the formula (main PA area × main PA velocity time integral × heart rate), PA accelerating time (PAAT), the systolic excursion of the tricuspid annular plane (TAPSE) and right ventricular wall thickness at end-diastole (RVWTd) were assessed. All echocardiographic measurements were performed by an experienced examiner.

### Tissue preparation

Following the hemodynamic studies, the rats were mechanically ventilated via a tracheostomy and lung tissue was collected for quantitative real time-polymerase chain reaction (right upper lobe) and Western blot analysis (right middle lobe). Thereafter, the lung was perfused through a PA cannula with phosphate-buffered saline and fixed by 4% paraformaldehyde for immunohistochemistry or injected with barium/gelatin mixture for morphometric analysis [24]. The lung tissue was subsequently stained with elastica van Gieson staining for vascular morphometry as described previously [12, 24]. Right ventricular hypertrophy (RVH) was assessed using Fulton`s index (right ventricle [RV]/left ventricle [LV] plus septum [S]). Both the right and left ventricles were prepared for histological analysis by Masson's trichrome staining or elastica van Gieson staining.

### Pulmonary artery morphometric analysis

All the barium-filled PAs in each lung section were examined at × 400 using a light microscope (Olympus CX33, Tokyo, Japan), as previously described [24]. The PAs were categorized structurally as either muscularized described by the presence of muscle (complete medial coat, incomplete medial coat, up to 25% of muscle being present) or non-muscular (with no muscle present). The percentage of muscularized arteries in peripheral pulmonary arteries (% muscularization) with an external diameter between 15 and 50 μm was calculated. The medial wall thickness (distance between external and internal elastic laminae) was measured along the shortest curvature, and the percent medial wall thickness (%MWT) as previously described [12, 22] in vessels measuring 15–50 μm, 50–100 μm and 100–200 μm separately. The number of distal PA with an external diameter between 15 and 50 μm and alveoli were counted in randomly chosen 10 fields of microscopic images (× 100) and the ratio of the number of distal PA to 100 alveoli was calculated. The morphometric analyses were performed by two independent examiners, blinded to the rat genotype and treatment group.

### Histological analysis of myocardium

Sections of myocardial tissue were stained with Mason-trichrome method. Fibrotic area in the RV and LV myocardium was quantified using ImageJ ver.1.51, as previously described [25]. For the quantification of myocardial fibrosis, average percentage of the fibrosis area were calculated using randomly selected 10 fields of microscopic images of the RV myocardium for each animal. For the assessment of vessel number in the myocardium, the sections were stained with elastica van Gieson staining for vascular morphometry. Intramyocardial small arteries (50–250 µm) and arteriole (10–50 µm) were counted. To assess the intramyocardial small arteries, all vessels in the tissue section were counted for each animal. For the intramyocardial arterioles, vessels were counted in 10 high power field at × 400 and mean number per field was evaluated.

### Immunohistochemistry

Immunohistochemistry was conducted as previously described [24, 26, 27]. Paraformaldehyde fixed paraffin sections were deparaffinized and rehydrated. Epitope retrieval was performed by boiling the sections in citrate buffer (pH 6.0) for 20 min. After washing, the sections were incubated with 0.3% hydrogen peroxide in methanol for 30 min in order to block endogenous peroxidase. 1% bovine serum albumin in phosphate buffered saline was used as the blocking buffer for 1 h. Sections were then incubated with primary antibodies overnight at 4 ℃. After streptavidin–biotin amplification (LSAB2 kit, DAKO, Carpinteria, CA), the slides were incubated with 3, 3’-diaminobenzidine and counterstained with hematoxylin. Thereafter, dehydralization with ethanol and xylene was performed before mounting on slides with mounting media. A negative control was performed using isotype-matched mouse IgG or rabbit immunoglobulin (DAKO), instead of the primary antibody. Primary antibodies were as follows: anti-macrophage/monocyte (1:200 dilution, mouse monoclonal, clone ED-1, MAB1435, Sigma); anti-BMPR2 (1:200 dilution, mouse monoclonal 3F6F8, Thermo Fisher Scientific); anti-α-smooth muscle actin (α-SMA, 1:1000 dilution, mouse monoclonal 1A4, DAKO); anti-Ki67 (1:200 dilution, rabbit monoclonal ab92742 Abcam, Cambridge, UK); anti-smooth muscle myosin heavy chain-SM2 (1:400 dilution, mouse monoclonal 7601, Yamasa); anti-nonmuscle myosin heavy chain-SMemb (1:1000 dilution, mouse monoclonal 7602, Yamasa); anti-Id1 (1:50 dilution, mouse monoclonal, sc-133104, Santa Cruz Biotechnology, Dallas, TX); anti-PDE type 5 (1:50 dilution, rabbit polyclonal, ab14672, Abcam). Quantitative analysis for ED-1 immunohistochemistry was performed by counting the total number of ED-1 positive cells per 30 high power field which were randomly selected [24]. The quantification for Ki67 was performed by determining the number of muscularized vessels with Ki67 positive staining in the intima-media per slide as previously described [26]. Semi-quantitative analysis for staining of α-SMA, SM-2 and SMemb was performed by grading the extent of intensity of the area of staining in the intima-media of the vessels graded as: 0- no staining, 1- mild staining up to 25%, 2- moderate of 25%–50% proportion of the intima media and 3- severe was above 50% as previously described [24, 28]. The localization and intensity of immunoreactivity were assessed by two independent examiners, blinded to the rat genotype and treatment group.

### Immunofluorescent staining

Lung sections were incubated with primary antibodies that recognize Ki67 (1:200 dilution, Abcam) and α-SMA (1:1000 dilution, DAKO) overnight at 4 °C. This was then followed by incubation with Alexa 488 (goat anti-rabbit) and Alexa 594 (goat anti-mouse)-conjugated secondary antibodies (Molecular Probes, Eugene, OR). Vessels were assessed using confocal microscopy (FV1000, Olympus).

### Quantitative real time polymerase chain reaction

Total RNA was extracted and purified from whole lung tissue using Trizol® reagent (Life Technologies). The quantity and quality of RNA was performed and assessed with Nanodrop 2000 (Thermo Fisher Scientific). Quantitative real-time PCR was performed using a StepOnePlus Real Time PCR System with TaqManR Gene Expression Assays on Demand probes (Rn01437214_m1[*Bmpr2*]*,*Rn01639345_m1 [*Pde5a*], Rn00561137_m1 [*Ednra*], Thermo Fisher Scientific). Relative standard curve values were determined with StepOne software (Applied Biosystems), and values were normalized against ß actin. Data are expressed as fold-change compared with the control group.

### Western blot

The collected lung tissues were kept in liquid nitrogen when transferring to -80ºC freezer before being homogenized and lysed with RIPA lysis buffer (ULTRARIPA® kit for Lipid Raft, BioDynamics Laboratory, Tokyo, Japan) followed by the detection of protein concentration using the BCA assay kit (Thermo Fisher Scientific). Then, equal amounts of samples were subjected to 10% SDS–PAGE, and the separated proteins were electro-blotted onto 0.2 μm PVDF membranes (BioRad, Hercules, CA), which were blocked with 5% skim milk in TBST for 2 h. The membranes were then incubated at 4 ℃ overnight with respective primary antibodies and the following day with corresponding secondary antibodies. Antibodies bound to protein blots were detected using Western Lightening Chemiluminescence Reagent Plus (Perkin Elmer Life Science, Boston, MA, USA), visualized on ImageQuant LAS-4000 mini (FUJIFILM) and the expression was quantitatively analyzed by densitometry measurements using ImageJ ver.1.51 software.

The primary antibodies used are as follows: anti-BMPR2 (1:500 dilution, 612292, BD Biosciences, San Jose, CA); anti-phospho SMAD1/5/9 (1:1000 dilution, Ser463/465, Cell Signaling Technology, Danvers, MA); anti-SMAD1/5/9 (1:1000 dilution, ab80255, Abcam); anti-phospho-Akt (1:5000 dilution, Ser473 [D9E] XP, Cell Signaling Technology); anti-Akt (pan) (1: 5000 dilution, C67E7, Cell Signaling Technology); anti-phospho SMAD2/3 (1:2500 dilution, #8828, Cell Signaling Technology); anti-SMAD2/3 (1:2500 dilution, #8685, Cell Signaling Technology); anti-β-Actin (1:200,000 dilution, Sigma Aldrich); anti-α-SMA (1:1000 dilution, mouse monoclonal 1A4, DAKO); anti-smooth muscle myosin heavy chain-SM2 (1:2000 dilution, mouse monoclonal 7601, Yamasa). The respective secondary antibodies were anti-mouse IgG horseradish peroxidase linked (HRP) and anti-rabbit IgG-HRP (dilution 1: 200,000 Santa Cruz Biotechnology).

### Pulmonary artery tension measurements

A set of rats (n = 6) were assigned for tension measurement studies and MCT (60 mg/kg, Sigma Aldrich) was injected subcutaneously. On day 21 after MCT injection, rats were anesthetized with intraperitoneal pentobarbital (50 mg/kg). The lungs and heart were removed en bloc. The first-order intra-pulmonary artery were isolated and gently cleaned of fat and connective tissue. The changes in isometric force were measured with a force–displacement transducer (TB-651 T; Nihon Kohden, Tokyo, Japan) connected to a carrier amplifier (EF-601G; Nihon Kohden) and were recorded on a pen recorder (WT-645G; Nihon Kohden). Ring segments (length: 2 mm) were cut and suspended vertically between hooks in organ baths (20 mL) containing modified Krebs–Henseleit (KH) solution, which was maintained at 37 ºC and bubbled with a mixture of 95% air-5% CO2. After each ring was equilibrated for 40–60 min at an optimal resting tension (0.75 g for both WT and +/44insG rats), the ring was contracted with 70 mM KCl to obtain the active tension [29]. After 25 min, the ring was washed with modified KH solution and repeated every 10–15 min to allowed to equilibrate. A cumulative concentration–response curve to prostaglandin F2α (PGF2α, 10^–8^–10^–5^ M, Maruishi Pharmaceutical Co., Ltd., Osaka, Japan) was obtained. With most preparations, vasocontraction responses were repeated with other vasocontraction agents, phenylephrine (10^–8^–10^–5^ M, Kowa Co., Ltd., Nagoya, Japan) and endothelin-1(ET-1, 10^–10^–10^–8^ M, Enzo Life Sciences, Farmingdale, NY) in different time series. 70 mM KCl-induced contraction was taken as 100% in these contracting responses. To investigate the response to a vasorelaxing agent, the rings were precontracted with PGF2α (10^–6^–10^–5^ M based on the PGF2α concentration–response curve) to obtain 50–85% of the maximal contraction induced by 70 mM KCl. After precontraction with PGF2α, a cumulative concentration–response curve was obtained for acetylcholine (Ach, 10^–8^–10^–4^ M, Nacalaitesque, Kyoto, Japan) by producing a stepwise increase in the Ach concentration as soon as a stable response was reached to each preceding level. Finally, 10^–4^ M papaverine (Nacalaitesque) was added to produce maximal relaxation. Papaverine-induced relaxation was taken as 100%. Vasorelaxation responses were repeated with another vasocontraction agent sodium nitroprusside (SNP, 10^–9^–10^–5^ M, Nacalaitesque) in different time series.

### Statistical analysis

Data are presented as means ± standard errors of means. Comparisons between the groups were made using, two-tailed unpaired *t*-test, one-way analysis of variance (ANOVA) with Bonferroni's multiple comparisons, repeated measures (two-way) ANOVA with Holm method for the vascular tension measurements of PA rings or log-rank test using Kaplan–Meier curves for survival analysis, as appropriate. JMP version 14 (SAS institute Inc., Cary, NC) was used for the vascular tension measurements and GraphPad Prism version 7.02 (GraphPad Software, San Diego, CA) was used for the rest of the experiments. A p value < 0.05 was considered statistically significant.

## Results

### Generation of rats with CRISPR/Cas9-mediated *Bmpr2 *mutation

Using CRISPR/Cas9 and TAKE method, a single nucleotide, guanine, was inserted in exon 1 of *Bmpr2 *at the position between 43 and 44 relative to the translation start site (Fig. 1B). The mutation, the only one we obtained in the founder rats, predicted to cause a frameshift at amino acid 15 (Leu15Cys) and premature termination of *Bmpr2* translation. In fact, a *Bmpr2* mutation (c.44delC, p.15 fs) that causes a frameshift in exon 1 similar to the mutation site generated in this study was found in heritable PAH patients [30].

To examine whether the generated *Bmpr2 *mutation, referred to as “44insG” in this study, has a functional role in the early stages of embryonic development, as shown in mice with deficient *Bmpr2* [31], we examined the outcomes of breeding patterns when heterozygous rats were crossed between heterozygous rats ([+/44insG] × [+/44insG]) and when heterozygous rats were crossed with wild-type rats ([+/44insG] × [+/+]). Crossing of both heterozygous rats resulted in a 1:2:0 ratio ([+/+]: [+/44insG]: [44insG/44insG]) in the offspring, as opposed to the expected 1:2:1 ratio. Thus, 25% of expected rats of genotype 44insG/44insG could have died in utero due to embryonic lethality. Crossing between heterozygous *Bmpr2 *rats and wild-type resulted in 1:1.13 ratio ([+/+]:[+/44insG]) outcome in the offspring which was close to the 1:1 ratio expected (Table 1).

**Table 1:** Genotypes of the offspring from the intercross between the heterozygous rats ([+/44insG] and [+/44insG]) and between the heterozygous and wild-type ([+/44insG] and [+/+])

	Cross: (+/44insG) × (+/44insG)			Cross: (+/44insG) × (+/+)		
Genotype	Number	Observed %	Expected %	Number	Observed %	Expected %
+/ +	8	33.3	25	24	47.1	50
+/44insG	16	66.7	50	27	52.9	50
44insG/44insG	0	0	25	0	0	0
Total	24	100	100	51	100	100

+ , wild-type allele; 44insG, mutation allele in *Bmpr2*, a single nucleotide (guanine) insertion in exon 1 of *Bmpr2* at the position between 43 and 44 relative to the translation start site

BMPR2 and phosphorylation of the canonical and non-canonical downstream proteins, SMAD1/5/9 and AKT were reduced significantly in the lungs of +/44insG rats compared with WT at baseline (Fig. 1C–D and Additional file 1: Figure S1A–D, p < 0.001). Id1 immunoreactivity was observed in a few vessels of the lung in the WT animal, while no immunoreactivity was observed in the mutant rats (Additional file 1: Figure S1I and J). However, there was no difference in phosphorylated SMAD2/3 levels in the lungs. With echocardiography performed at 7 weeks of age, there were no significant difference in both LV and RV function determined by LV ejection fraction and TAPSE (Additional file 1: Table S1). We subjected both the +/44insG rats and WT littermates to the different study groups shown in the Figs. 1E–I.

### Rats with the *Bmpr2* mutation exhibit a similar phenotype at 3 weeks after MCT injection

**Fig. 2:**
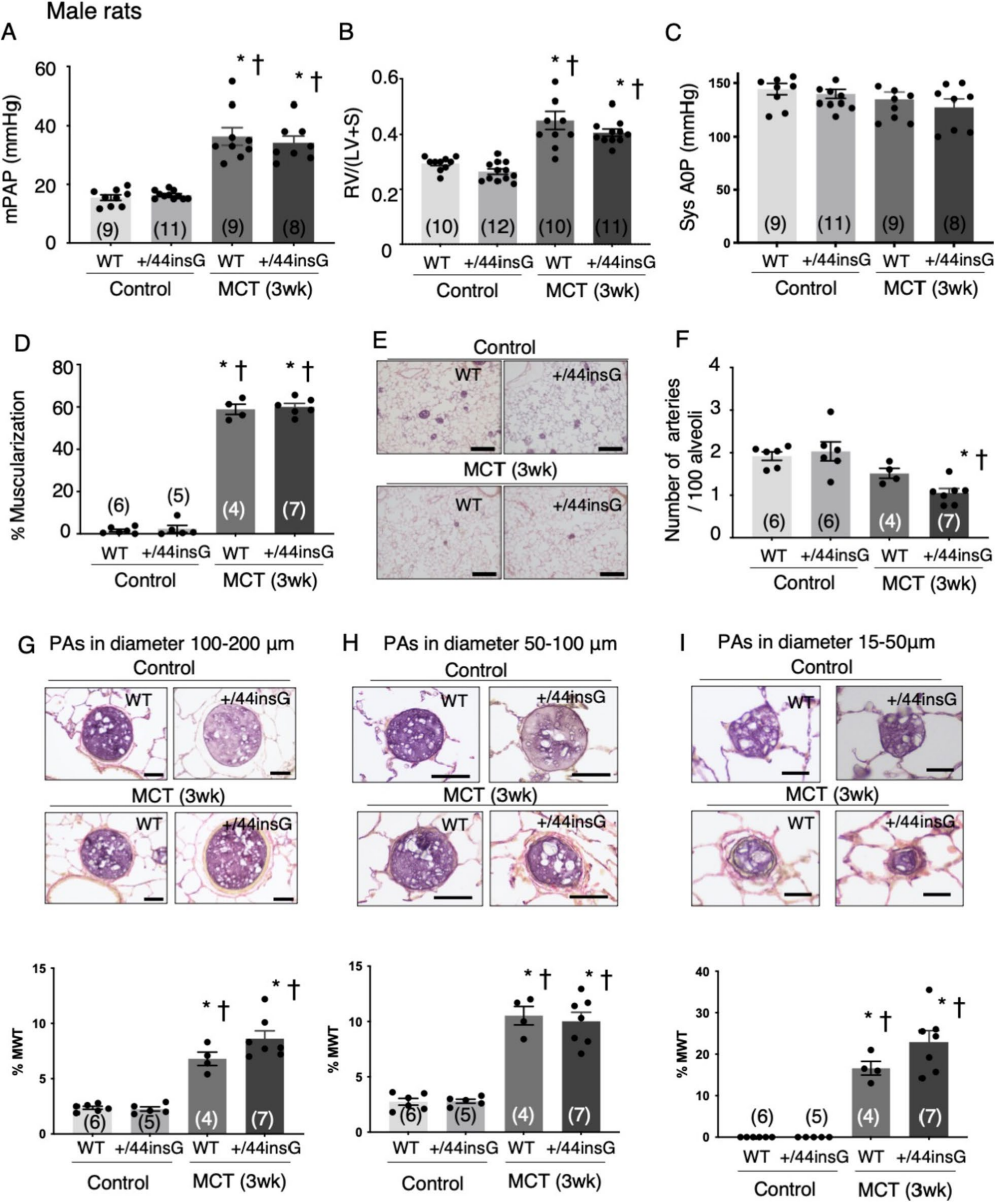
*Bmpr2* mutant rats are phenotypically similar to wild-type rats at 21 days after MCT injection. All the measurements are done in male rats with wild-type (WT) and *Bmpr2* mutation (+/44insG) at 3 weeks after monocrotaline (MCT [3 wk]) or saline injection (Control). **A** Mean pulmonary artery pressure (mPAP) (n = 37, WT and +/44insG), **B** the weight ratio of right ventricle/left ventricle plus septum (RV/[LV + S]) (n = 43 WT and +/44insG) and **C** systolic aortic pressure (Sys AOP) (n = 37, WT and +/44insG) at 3 weeks after MCT or saline injection. **D** The quantification of percentage muscularisation of distal PAs (n = 21, WT and +/44insG). **E** and **F** Representative images of the distal PAs and the quantification of the number of distal PAs per 100 alveoli (n = 23, WT and +/44insG) assessed in control and MCT-treated rats. Scale bar: 200 μm (**G**–**I**) Representative images of barium filled PAs with diameter 100–200 μm (**G**), 50–100 μm (**H**), 15–50 μm (**I**) in control (top images) and MCT-treated rats at 3 weeks (bottom images) and the quantification of medial wall thickness (MWT) (n = 22, WT and +/44insG). Scale bar: 50 μm (**F** and **G**), 25 μm (**H**). The numbers in parentheses represent the numbers of rats examined. Data are presented as means ± SEM; one-way ANOVA followed by Bonferroni’s multiple comparison test; *: P < 0.01 vs Control, WT; †: P < 0.01 vs Control, +/44insG. mPAP = mean pulmonary artery pressure; RV/(LV + S) = weight ratio of right ventricle/left ventricle plus septum; Sys AOP = systolic aortic pressure; WT = wild-type; +/44insG = rats with bone morphogenetic protein receptor type 2 mutation; MCT = Monocrotaline; PAs = Pulmonary arteries; %MWT = percentage of medial wall thickness; wk = weeks; mPAP = mean pulmonary artery pressure; RV/(LV + S) = weight ratio of right ventricle/left ventricle plus septum; WT = wild-type; +/44insG = rats with bone morphogenetic protein receptor type 2 mutation; MCT = Monocrotaline; PAs = Pulmonary arteries; %MWT = percentage of medial wall tension; wk = weeks

Significant PH was induced in WT and +/44insG male rats 3 weeks after MCT injection compared with saline injected controls with no significant differences between WT and +/44insG in mean PAP, RV/(LV + S) and systolic AOP (Fig. 2A–C, Additional file 1: Table S2). By lung histological analysis, the muscularization of distal PA was induced in MCT-injected animals with no significant difference between WT and +/44insG rats (Fig. 2D Additional file 1: Figure S2 F-I). The number of distal PAs per 100 alveoli was lower in +/44insG rats 3 weeks after MCT than control rats, however, it did not differ between WT and +/44insG rats (Fig. 2E and F). % MWT was higher in MCT-injected animals with no significant difference between WT and +/44insG rats (Fig. 2G–I). Immunohistochemistry using the ED-1 macrophage/monocyte marker revealed that both WT and +/44insG rats have increased ED-1 positive cells in the lungs of MCT injected rats with no significant differences between WT and +/44insG rats (Additional file 1: Figure S2A–E). Myocardial histology, fibrotic area and number of intramyocardial vessels in LV and RV in rats 3 weeks after MCT, did not differ between WT and +/44insG rats (Additional file 1: Figure S3).

In the female rats evaluated at 3 weeks after MCT injection (n = 23), only modest PH was induced with no significant difference between both WT and +/44insG rats (Additional file 1: Figure S4A and B, Table S3), although there was a significant increase in % muscularization, %MWT, decrease of the number of distal PA after MCT injection (Additional file 1: Figure S4 D and E, Table S3).

### Assessment at 6 months of age and chronic hypoxia model using male rats

Since previous studies with *Bmpr2* mutant rats have reported conflicting results regarding spontaneous PH [18, 19], we next evaluated male rats at 6-months of age. There was no development of spontaneous PH with no significant difference between WT and +/44insG rats in pulmonary hemodynamics and lung histology (Additional file 1: Figure S5, Table S4). In the male rats exposed to chronic hypoxia for 3 weeks (n = 11), there were also no obvious differences in PH and cardiac function between WT and +/44insG rats, although %MWT of the distal PA was higher in the +/44insG than WT animals (Additional file 1: Figure S6, Table S5).

### *Bmpr2* mutation reduces survival with the exacerbation of late pulmonary vascular disease

**Fig. 3:**
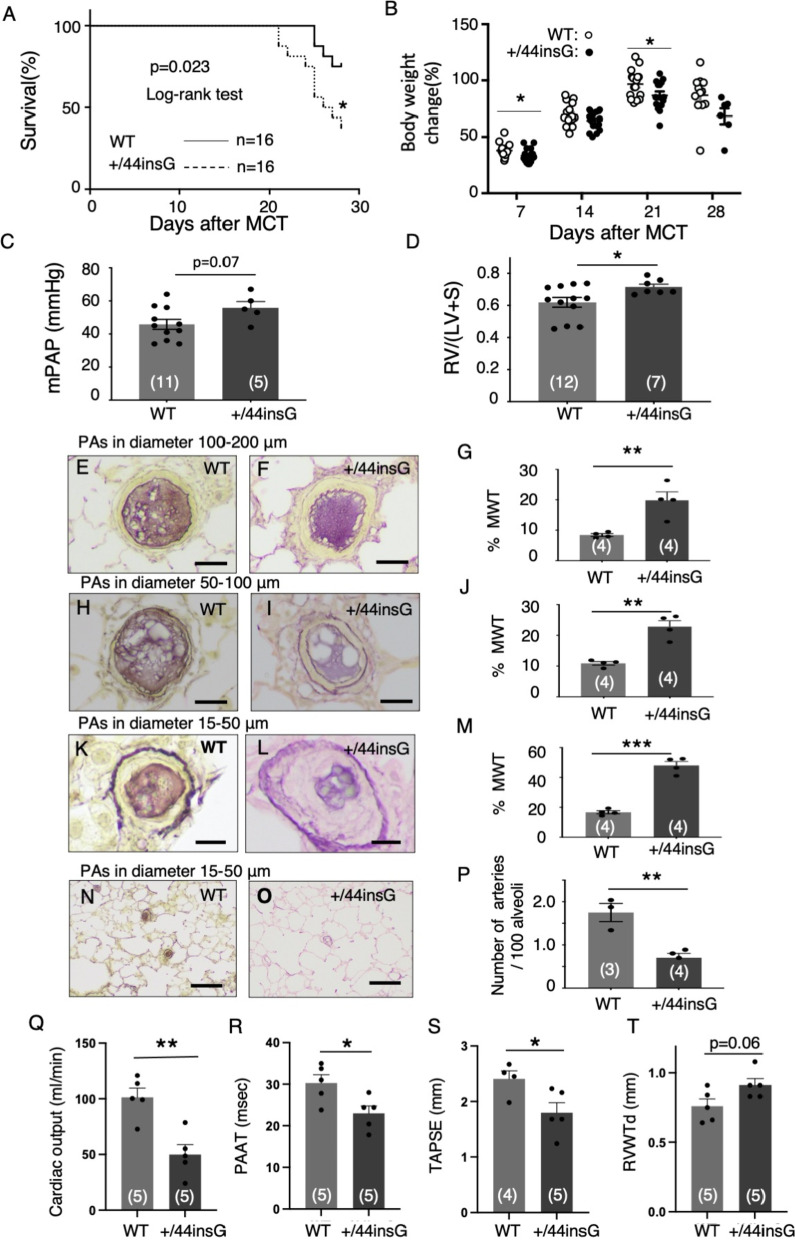
Reduced survival in male rats with *Bmpr2* mutation after MCT injection. **A** Survival outcomes of wild-type (WT) and *Bmpr2* mutation (+/44insG) rats after monocrotaline (MCT) injection. **B** The body weight percentage increment from start to the end of the experiment in WT (open circle) and +/44insG (closed circle) rats (n = 32, WT and +/44insG). **C** Mean pulmonary artery pressure (mPAP) of rats surviving on day 28 after MCT injection (n = 16, WT and +/44insG). **D** The weight ratio of right ventricle/left ventricle plus septum (RV/[LV + S]) (n = 19). **E**–**M** Representative images of barium filled proximal PAs with diameter size 100–200 μm (**E–G**), 50–100 μm (**H**–**J**), 15–50 μm (**K**–**L**) and the quantification of medial wall thickness (%MWT) at 4 weeks after MCT injection for WT and +/44insG respectively (n = 8, WT and +/44insG). Scale bar: 50 μm (**E** and **F**), 25 μm (**H** and **I**), 10 μm (**K** and **L**). **N**–**P** Representative images indicating the number of barium filled distal PAs and the quantification of the distal PAs per 100 alveoli at 4 weeks after MCT injection in WT and +/44insG respectively (n = 7, WT and +/44insG). Scale bar: 50 μm. **Q** Measurement of cardiac output, (**R**) pulmonary artery acceleration time (PAAT), **S** tricuspid annular plane systolic excursion (TAPSE), and (**T**) right ventricular wall thickness in diastole (RVWTd) in WT and +/44insG rats 24–26 days after MCT treatment (n = 10, WT and +/44insG). The numbers in parentheses represent the numbers of rats examined. For panel **A**, data are presented by Kaplan–Meier survival plot; for panel **B** data are presented as scatter plots indicating all data points; for panels **C**, **D**, **G**, **J**, **M** and **P**, the data are presented as means ± SEM; Log-rank (Mantel-Cox) test for panel **A**, unpaired t-test for panels **B**–**D**, **G**, **J**, **M**, **P** and **Q**–**T**; *p < 0.05, **p < 0.01, ***p < 0.001. WT = wild-type; +/44insG = rats with bone morphogenetic protein receptor type 2 mutation; MCT = monocrotaline; mPAP = mean pulmonary artery pressure; RV/(LV + S) = weight ratio of right ventricle/left ventricle plus septum; PAs = pulmonary arteries; %MWT = the percentage of medial wall thickness; PAAT = pulmonary artery acceleration time; TAPSE: tricuspid annular plane systolic excursion; RVWTd: right ventricular wall thickness in diastole

Next, we investigated the impact of the *Bmpr2 *mutation 44insG on survival of rats after MCT injection. The survival was significantly reduced in +/44insG rats than WT (75% in WT vs 38% in +/44insG at day 28, p < 0.05, Fig. 3A). Consistent with worse survival outcome, percentage of the body weight gain from initiation of experiment on day 7 and day 21 of MCT injection were significantly reduced in +/44insG rats compared to WT (p < 0.05, Fig. 3B). Analysis among the animals surviving at day 28, RV/(LV + S) was significantly higher in the +/44insG mutant rats than WT littermates (p < 0.05, Fig. 3D, Additional file 1: Table S6). Even though the mPAP and RVSP were not significantly different between the groups, presumably owing to the fewer number of surviving mutant rats for analysis (n = 11 vs 5 for mPAP and RVSP), they were on the higher side than the WT littermates (p = 0.07, Fig. 3C, p = 0.16, Additional file 1: Figure S7B). AOP and hematocrit did not show significant differences between the two groups (Additional file 1: Figure S7A and C). By lung histological analysis, the muscularization was observed in all the distal PAs in both groups (data not shown). The %MWT for vessels of all sizes (100–200, 50–100 and 15–50 μm) were significantly higher in +/44insG rat than in the WT littermates (p < 0.01, p < 0.01 and p < 0.001, respectively, Fig. 3E–M). Furthermore, number of small distal PAs were significantly fewer in +/44insG rat than in the WT littermates (p < 0.01, Fig. 3N–P). Cardiac output, PAAT and TAPSE determined by echocardiography at 24–26 days after MCT injection were significantly reduced in the +/44insG rats compared with WT (Fig. 3Q–T). There was no obvious RV myocardial interstitial fibrosis in control rats and 4 weeks after MCT injection with no significant differences between WT and +/44insG rats (Additional file 1: Figure S7D–I).

### Impaired PA contraction and increased proliferation of medial PASMC in *Bmpr2* mutant rats

**Fig. 4:**
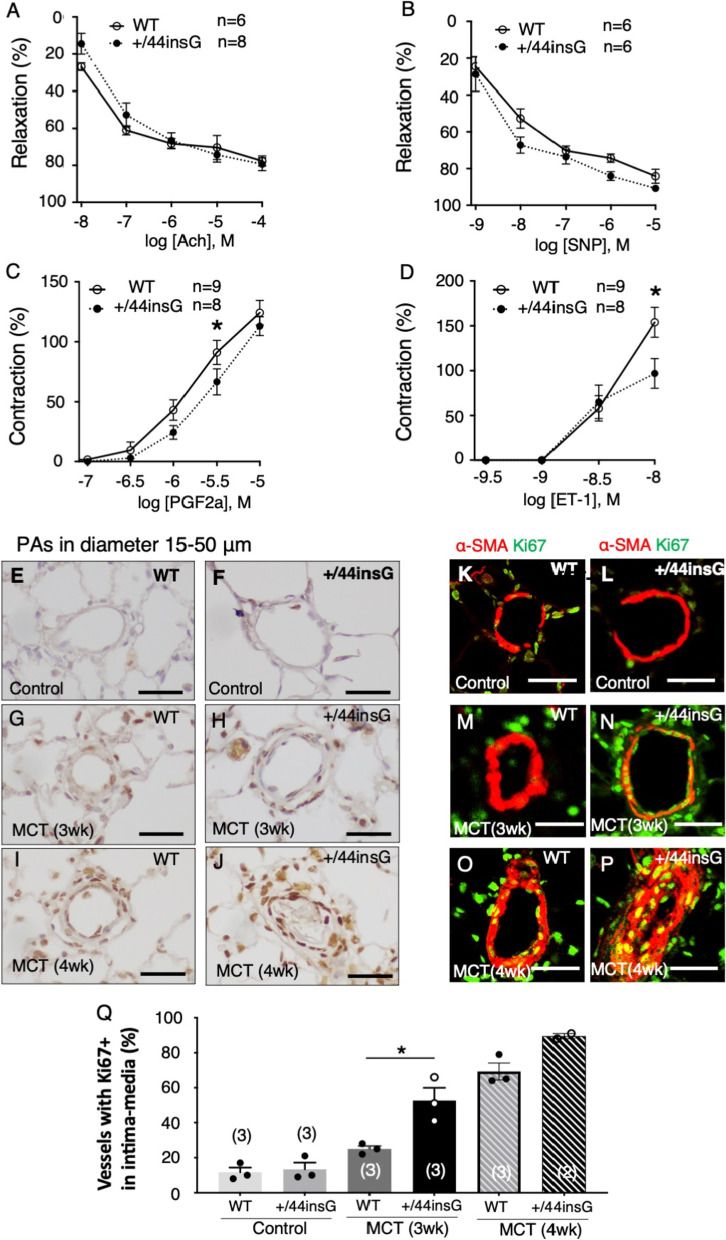
Impaired contraction of intra-pulmonary arteries and increased proliferation of medial smooth muscle cells in the lung from the male *Bmpr2* mutant rats. **A**–**D** Contraction and relaxation response to the vasoreactive agents were assessed using intrapulmonary artery (IPA) obtained from wild-type (WT, open circle) and *Bmpr2* mutation (+/44insG, closed circle) rats at 3 weeks after monocrotaline (MCT) injection. **A** and **B** The dose–response curve showing the relaxation of IPA to acetylcholine (Ach) (n = 14 WT and +/44insG) and sodium nitroprusside (SNP) (n = 12 WT and +/44insG). 10–4 M papaverine relaxation response was taken as 100%. **C** and **D** The dose–response curve showing the contraction of IPA to prostaglandin F2α (PGF2α) (n = 17 WT and +/44insG) and endothelin-1 (ET-1) (n = 17 WT and +/44insG). 70 mM KCl contracting response was taken as 100%. **E-J** Representative images for Ki67 immunohistochemistry in distal PAs in control rats assessed at 3 weeks after saline injection, 3 weeks or 4 weeks after MCT injection. Scale bar: 25 μm. **K**–**P** Representative immunofluorescent images of distal PAs stained for Ki67 (green) and α-SMA (red) in control, 3 weeks or 4 weeks after MCT injection. Scale bar: 25 μm. **Q** Quantitative analysis of vessels with Ki67 + cells in the distal PAs in control animals, 3 weeks and 4 weeks after MCT injection respectively (n = 17 WT and +/44insG). The numbers in parentheses represent the numbers of rats examined. For panels **A**–**D**, data are presented by points and connecting lines with error bars; for panel **Q**, the data are presented as means ± SEM; repeated measures (two-way) ANOVA with Holm method was used for panels **A**–**D**; one-way ANOVA followed by Bonferroni’s multiple comparison test was used for panel **Q**. *p < 0.05, **p < 0.01. WT = wild-type; +/44insG = rats with bone morphogenetic protein receptor type 2 mutation; Ach = acetylcholine; SNP = sodium nitroprusside; PGF2α = prostaglandin F2α; ET-1 = endothelin-1; PAs = pulmonary arteries; Day = days after monocrotaline injection; α-SMA = α-smooth muscle actin; wk = weeks; PAs pulmonary arteries

To assess the vasorelaxation responses of PA, acetylcholine (Ach) and sodium nitroprusside (SNP) induced dose-dependent relaxation of intrapulmonary artery (IPA) rings from the WT and +/44insG groups were examined. IPA showed no significant differences in the relaxation response to Ach and SNP in the two groups (Fig. 4A and B). Vasoconstriction induced by 10^–5.5^ M of PGF2α and 10^−8^ M of ET-1 was significantly reduced in +/44insG rats compared with WT (p < 0.05, Fig. 4C and D). Endothelin receptor type A (ET-A) mRNA levels, as determined by real-time qPCR, were lower in +/44insG rats compared with WT only in control animals, but was comparable between WT and +/44insG rats at 3 or 4 weeks after MCT injection (Additional file 1: Figure S8).

Next, we investigated the proliferation of the cells in the lung by immunostaining using Ki67 antibody. There were few Ki67+ cells in the PAs in the control rat in the both groups (Fig. 4E–F). At 3 weeks and 4 weeks after MCT injection, Ki67 + cells were observed in the media and adventitia of muscularized distal PAs suggesting increased cell proliferation (Fig. 4G–J). The number of the distal PAs with Ki67+ cells were significantly increased in the +/44insG rats 3 weeks after MCT compared with WT (Fig. 4Q, 11.7 ± 2.7% vs 13.3 ± 3.8 in control, P = 0.99; 25.0 ± 1.7% vs 52.7 ± 7.3 in 3 weeks after MCT, p < 0.01; 69.3 ± 4.8% vs 89.5 ± 1.5 in 4 weeks after MCT, p = 0.19). Immunofluorescent staining using α-smooth muscle action (SMA) and Ki67 antibodies revealed that increased Ki67+ cells in the distal PA were colocalized with α-SMA suggesting increased proliferation of PA smooth muscle cells (SMC) (Fig. 4K–P).

### Phenotypic modulation of medial PASMC in *Bmpr2* mutant rats

**Fig. 5:**
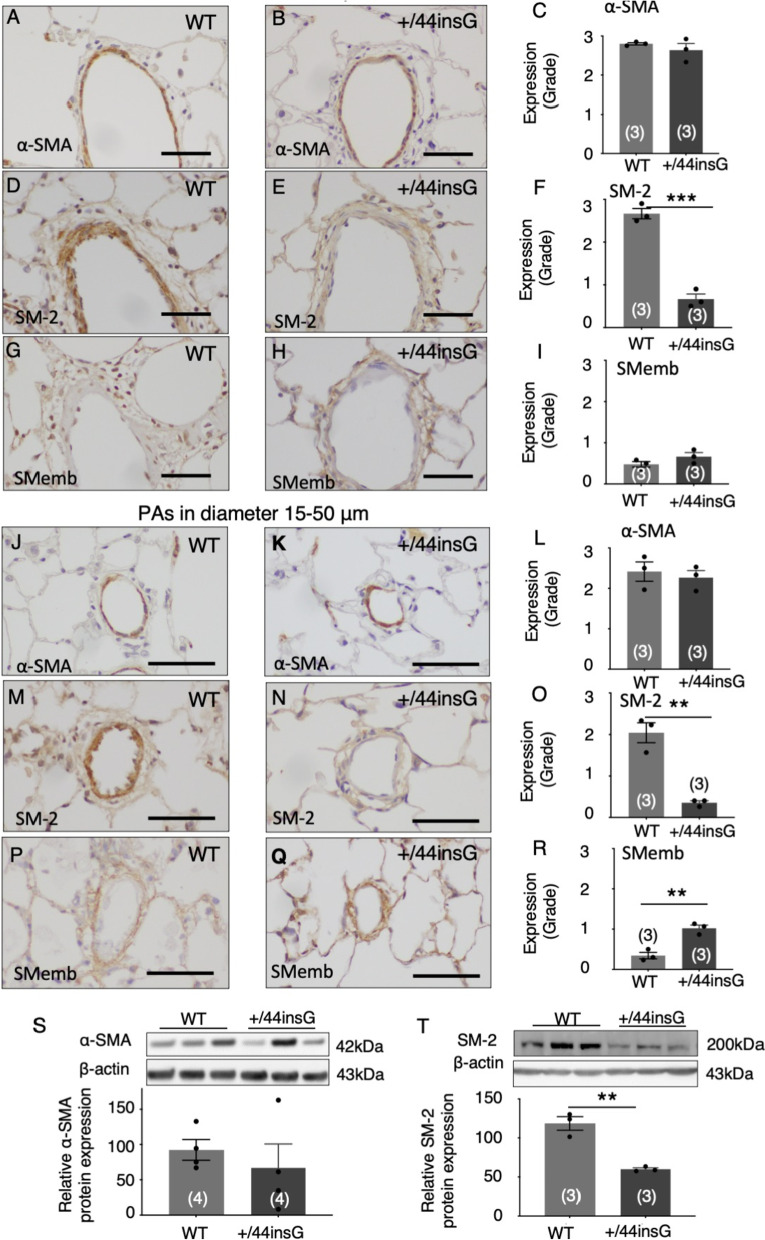
Phenotypic changes of medial smooth muscle cells in the lungs of rats with *Bmpr2* mutation. Immunohistochemical staining of the pulmonary arteries (PAs) in male rats at 3 weeks after monocrotaline injection using smooth muscle cell differentiation state specific markers, α-smooth muscle actin (SMA), smooth muscle myosin heavy chain SM-2 (SM-2, as a mature phenotypic marker) and nonmuscle myosin heavy chain SMemb (SMemb, as an immature phenotypic marker). **A**–**I** Representative immunohistochemistry images and semi-quantitative analysis indicated by grade for α-SMA, SM-2 and SMemb positive cells in proximal PAs in wild-type (WT) and *Bmpr2* mutant (+/44insG) rats, respectively (n = 6). Scale bar: 50 μm. **J**–**R** Representative immunohistochemistry images and semi-quantitative analysis indicated by grade for α-SMA, SM-2 and SMemb positive cells in distal PAs in WT and +/44insG respectively (n = 6). Scale bar: 50 μm. **S** and **T** Representative Western blots and the quantification of relative α-SMA (**S**, n = 4 for each group) and SM-2 (**T**, n = 3 for each group) protein expression in the lung tissue of WT and +/44insG rats respectively. The numbers in parentheses represent the numbers of rats examined. The data are presented as means ± SEM; unpaired t-test was used for analysis; **p < 0.01, ***p < 0.001. PAs = pulmonary arteries; WT = wild-type; +/44insG = rats with bone morphogenetic protein receptor type 2 mutation; α-SMA = α-smooth muscle actin; SM-2 = smooth muscle myosin heavy chain SM-2; SMemb = nonmuscle myosin heavy chain embryonic isoform

There are two types of smooth muscle phenotype: synthetic or contractile and the latter is important for the regulation of vascular tone. Given the observation suggesting impaired contraction of the PAs and proliferative state of PASMC in the PAs, we further examined the PASMC phenotype by immunostaining with differentiation state-specific markers to determine whether PASMCs are contractile or synthetic phenotype on day 21 of MCT. The +/44insG rat showed that both proximal and distal pulmonary vessels had significant reduction of SM-2 staining (Fig. 5E–F, N–O, p < 0.001 and p < 0.01) which is a highly specific marker for indicating contractile phenotype and is the first to be lost in the process of modulation of the phenotype to the immature type [28]. This was also confirmed by western blot in which there was significant downregulation in expression of SM-2 in the +/44insG rats (Fig. 5T, p < 0.01). Further, there was an upregulation of the immature marker SMemb in the distal small PAs in the +/44insG rats (Fig. 5Q–R, J–L, p < 0.01). The α-SMA, which is a marker for the presence of PASMCs, did not show any significant differences between WT and +/44insG rats (Fig. 5A–C). This was also confirmed by western blot as there were no significant differences in the relative protein expression of α-SMA between WT and +/44insG rats (Fig. 5S, p = 0.52).

### Reduced survival with tadalafil treatment in the *Bmpr2* mutant rats

**Fig. 6:**
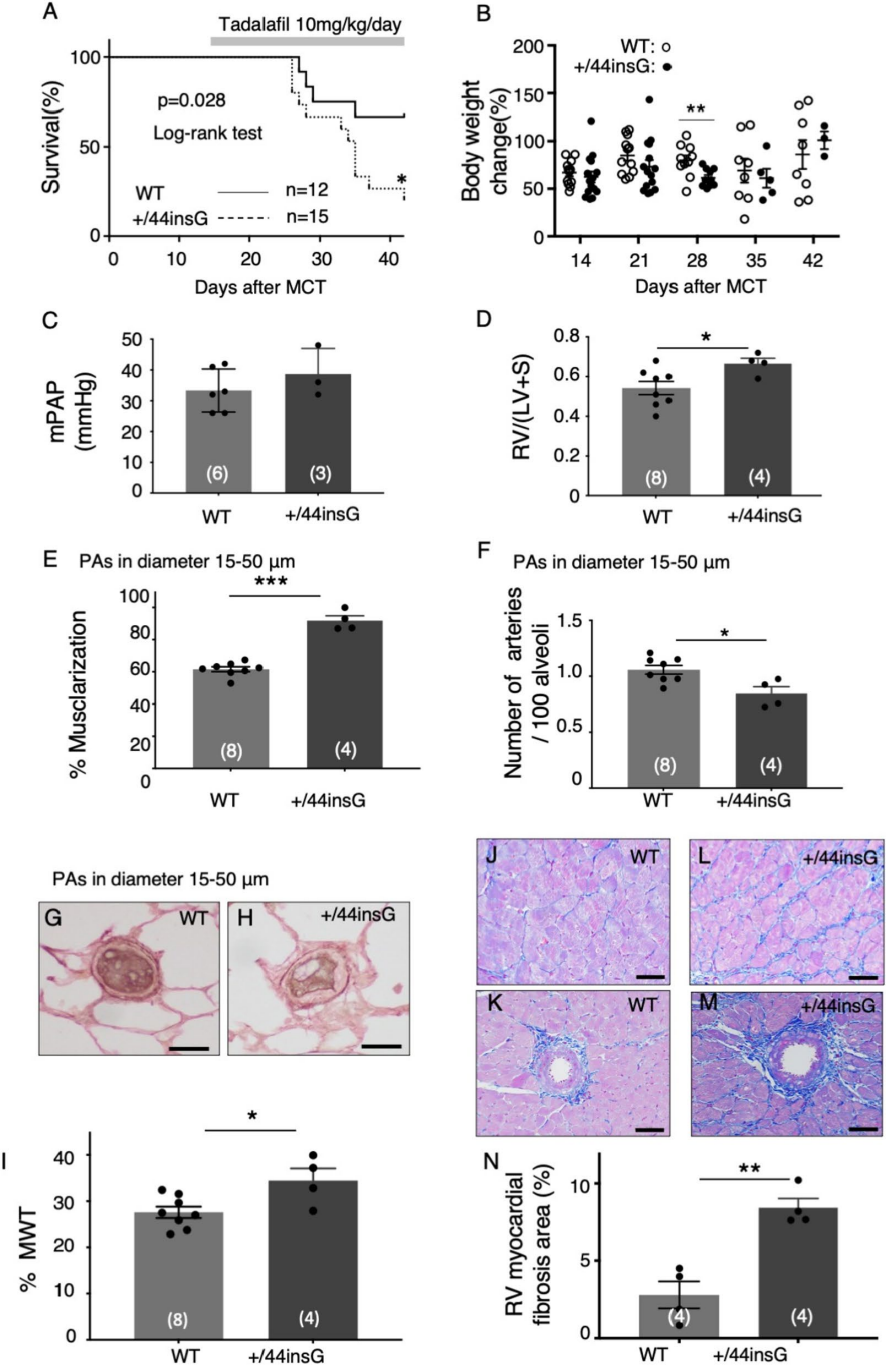
Efficacy of long-term tadalafil treatment from day 14 to 42 after MCT injection in *Bmpr2* mutant (+/44insG) and wild-type rats. **A** Survival outcomes of wild-type (WT) and *Bmpr2* mutation (+/44insG) rats after monocrotaline (MCT) injection treated with long-term tadalafil (10 mg/kg/day) from day 14 to 42 (n = 37, WT and +/44insG). **B** The percentage of body weight change from initiation of experiment in WT (open circle) and +/44insG (closed circle) rats. **C** mPAP (n = 9, WT and +/44insG) and **D** RV/(LV + S) (n = 12, WT and +/44insG). **E** The quantification of percentage muscularization of distal pulmonary arteries (PAs) (n = 12, WT and +/44insG) and **F** the quantification of the number of distal PAs per 100 alveoli (n = 12, WT and +/44insG). **G** and **H** Representative images of barium filled vessels in diameters 15–50 μm in WT and +/44insG rats respectively. Scale bar: 10 μm. **I** The quantification of the percentage of medial wall thickness (%MWT) in vessels sized 15–50 μm in WT and +/44insG rats (n = 12, WT and +/44insG). **J**–**M** Representative images of right ventricle myocardial tissue by Masson's trichrome stain and **N** the quantification of right ventricle myocardial fibrosis in WT and +/44insG at 6 weeks after MCT injection (n = 8, WT and +/44insG). Scale bar: 50 μm. The numbers in parentheses represent the numbers of rats examined. For panel **A**, data are presented by Kaplan- Meier survival plot and Log-rank (Mantel-Cox) test was used; for panel **B** data are presented as scatter plots indicating all data points; for panels **C**–**F**, **I** and **N**, the data are presented as means ± SEM; unpaired t-test for was used for analysis; *p < 0.05, **p < 0.01, ***p < 0.001. WT = wild-type; +/44insG = rats with bone morphogenetic protein receptor type 2 mutation; MCT = monocrotaline; mPAP = mean pulmonary artery pressure; RV/(LV + S) = weight ratio of right ventricle/left ventricle plus septum; PAs = pulmonary arteries; %MWT = the percentage of medial wall thickness; RV = right ventricle

In clinical practice, most PH patients present when the disease is advanced, and since we did not design our study along the preventive route of inhibiting disease progression, we decided to administer tadalafil at the time PH begins to become evident in the MCT model, but before decompression occurs. Tadalafil treatment in +/44insG and WT rats with MCT-PH for two weeks from day14 to 28 revealed no statistically significant differences in survival of animals, pulmonary hemodynamics, RVH and PVD (Additional file 1: Figure S10A–M, Table S7). There were no significant differences in RV myocardial interstitial and perivascular fibrosis at day 28 between WT and +44insG rats (Additional file 1: Figure S10N–R). Therefore, we next decided to subject the MCT-PH rats to a longer tadalafil treatment for 4-weeks. We assessed the survival with tadalafil treatment which was initiated at day 14 up to day 42 after MCT injection. Despite treatment with tadalafil, the +/44insG rats had a reduced survival compared with the WT littermates (67% in WT vs 20% in +/44insG at day 42, p < 0.05, Fig. 6A). Consistent with the worse outcome, +/44insG rats showed a lower trend in the percentage of the body weight gain from initiation of experiment compared with WT with significant differences on Day 28 after MCT (p < 0.01, Fig. 6B). Among the animals surviving at 6 weeks after MCT injection, RV/(LV + S) was significantly higher in +44insG rats compared with WT littermates (p < 0.05, Fig. 6D, Additional file 1: Table S8), while the mPAP, AOP and RVSP were not significantly different between the groups(Fig. 6C and Additional file 1: Figure S11A and B, Table S8). By lung histological analysis, %muscularization in the distal PAs was significantly higher in +/44insG rats than in the WT littermates (p < 0.001, Fig. 6E). The %MWT for vessels of size 15–50 μm were significantly higher in +/44insG rat than in the WT littermates (p < 0.05, Fig. 6G–I) while the proximal vessels 100–200 μm and 50–100 μm did not show any significant differences between the groups (Additional file 1: Figure S7D–F, G–I, p = 0.32 and p = 0.65). Furthermore, the number of small distal PAs were significantly fewer in +/44insG rats than in the WT littermates (p < 0.05, Fig. 6F). The percentage of RV myocardial interstitial fibrosis area was significantly higher in +/44insG rats than WT (Fig. 6N, 2.8 ± 0.9 vs8.4 ± 0.6%, p < 0.01). Notably, extensive RV myocardial fibrosis was observed in both interstitial and perivascular area in +/44insG rats (Fig. 6J–M). Phosphodiesterase type 5 (PDE5) mRNA levels, as determined by real-time qPCR, did not differ between WT and +/44insG rats at 3 or 4 weeks after MCT injection, although it was lower in both WT and +/44insG rats at 3 or 4 weeks after MCT injection compared to control rats (Additional file 1: Figure S9A). PDE5 immunoreactivity was observed in the hypertrophied medial wall of the PA in the rats 3 weeks after MCT injection (Additional file 1: Figure S9B).

## Discussion

**Fig. 7:**
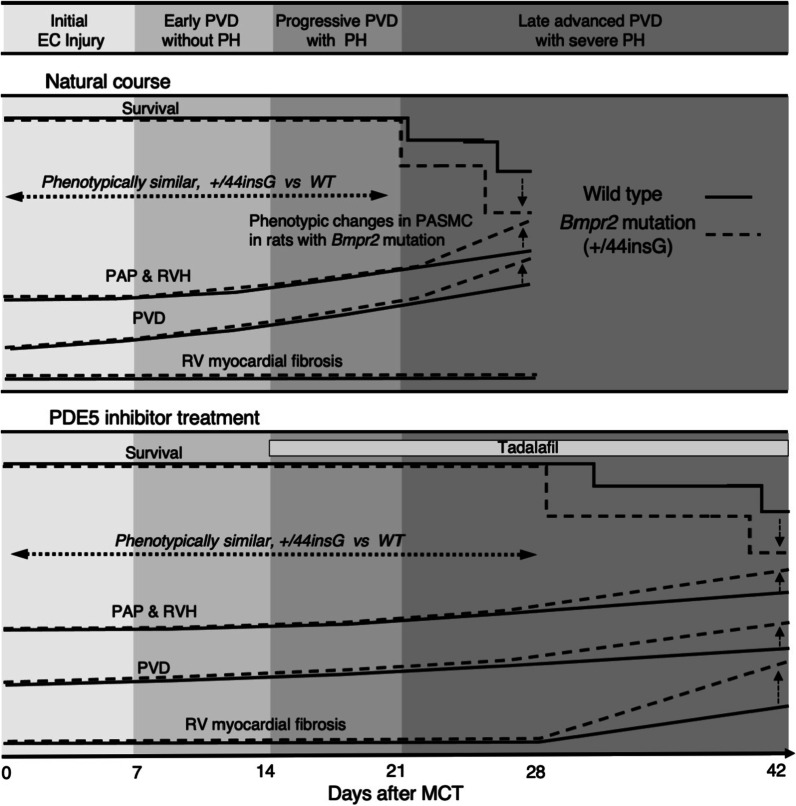
The impacts of *Bmpr2* mutation on the phenotype of rats with monocrotaline-induced pulmonary hypertension with or without phosphodiesterase type 5 inhibitor treatment. Schematic representation for the impacts of *Bmpr2* mutation on the course of monocrotaline (MCT) induced-pulmonary hypertension (PH) for the rats without treatment (top, natural course) and treated with a phosphodiesterase type 5 (PDE5) inhibitor (bottom) indicating survival, pulmonary vascular disease (PVD), pulmonary arterial pressure (PAP), right ventricular hypertrophy (RVH) and RV myocardial fibrosis in wild-type (WT, solid lines) and *Bmpr2* mutation (+/44insG, dashed lines) rats. Disease stage of MCT-PH is classified into initial endothelial cell (EC) injury from day 0–7 of MCT injection showing baseline survival, PVD, PAP and RVH; early PVD without pulmonary hypertension (PH) from day 7–14; progressive PVD with PH from day 14–21 showing increase in both PAP and RVH; advanced PVD with severe PH after day 21. EC = endothelial cell; PVD = pulmonary vascular disease; PH = pulmonary hypertension; BMPR2 = bone morphogenetic protein receptor type 2; +/44insG = the *Bmpr2* mutation generated in this study; WT = wild-type; PAP = pulmonary artery pressure; RVH = right ventricular hypertrophy; MCT = monocrotaline; PASMC = pulmonary artery smooth muscle cell; RV = right ventricle; PDE5 = phosphodiesterase type 5

The main findings of this study are: first, we have created for the first time, to the best of our knowledge, rats harbouring a monoallelic single nucleotide insertion in *Bmpr2* gene using a CRISPR/Cas9 genome editing which caused significant functional derangements in *Bmpr2* gene (i.e., reduced BMPR2 protein expression and SMAD1/5/9 phosphorylation in the lung, potential for developmental defect in embryonic or early postnatal period). Second, there was no functional and structural difference between WT and +/44insG male rats at 6 months of age and certainly in young animals 10 weeks of age as well as at 3 weeks after MCT injection. However, the mutation exacerbated obstructive PVD and worsened survival after 3 weeks post MCT injection. Third, the *Bmpr2* mutation confers phenotypic changes to the medial smooth muscle cells in the PAs of rats with established PH (i.e., transition to immature, hyperproliferative and less-contractile phenotype). Fourth, the mutant rats have reduced response to long-term pulmonary vasodilator treatment presumably by aggravation of right ventricular fibrosis as well as exacerbation of occlusive PVD (Fig. 7). These findings of the present study have clinical and mechanistic implications for the pathogenesis of PAH and may lead to the development of novel therapies to improve outcomes, especially for patients with heritable PAH (HPAH).

In the first study using rats with the monoallelic *Bmpr2* mutation in which 140 base pairs in the exon 1 were deleted by ZFN, Ranchoux et al. reported that the mutated rat developed PVD without PH at 3 months of life associated with endothelial-to-mesenchymal transition [32]. More recently, using rats with the *Bmpr2* mutation in which 16–140 base pairs in exon 1 or 527 base pairs in the intron and exon 1 boundary of the *Bmpr2* gene were deleted also using ZFN, spontaneous PH, impaired RV function and propensity to fatal PH due to inflammatory stimuli were shown in the mutated rats [18, 19]. We employed a newer technique [20] to create rats with a single point mutation in exon 1 of *Bmpr2* (referred to as +/44insG). As predicted, lung BMPR2 protein expression and both the canonical and non-canonical downstream signals were reduced in the +/44insG rats. Genotype analysis of the offspring in the (+/44insG) x (+/44insG) crossing revealed a 25% fatality of rats with biallelic 44insG mutation in embryonic stage as shown in the previous studies in mice [31]. Thus, the 44insG mutation generated in this study causes significant functional impairments of *Bmpr2* gene. Importantly, this is consistent with the initial report of a *Bmpr2* mutation (c.44delC, p.15 fs) found in HPAH patients that causes a frameshift in exon 1 similar to the mutation site generated in this study [30].

Genotype–phenotype correlation analysis have shown significant clinical phenotypic differences between *Bmpr2* mutation carriers and non-carriers [4–6, 33], highlighting the type of the mutation which affects clinical phenotype among the PAH patients with *Bmpr2* mutation. In clinical practice, about 24% are frameshift and 29% are nonsense *Bmpr2* mutations which contribute to the total 70% of all identified truncated mutations in HPAH that trigger nonsense-mediated mRNA decay (NMD) causing the degradation of the mutated mRNA and resulting into haploinsufficiency [34]. Other studies have shown that the patients with missense mutations had statistically significant worse phenotype and poor survival rates from diagnosis than those with truncating mutations [5]. Experimentally, significant PH with *Bmpr2* mutation have been demonstrated in transgenic mice expressing a dominant-negative *Bmpr2* gene in smooth muscle [35] and mice with endothelial-specific biallelic ablation of *Bmpr2* [36, 37], while mice with heterozygous *Bmpr2* mutation that cause a frameshift in the kinase domain have mild PH [31, 38]. These findings suggest that further mechanisms for differences in clinical outcomes associated with *Bmpr2* genotypes can be addressed by establishing rat models with other types of genetic alterations including missense or cell type-specific biallelic mutations.

In the present study, there was no obvious phenotypic difference between WT and +/44insG mutant rats at 3 weeks after MCT injection, 10 weeks and 6 months of age without MCT injection, and 3 weeks after hypoxic exposure. These results are consistent with a recent study showing that *Bmpr2* mutant rats were not significantly different in RVSP and RVH at 3 weeks after MCT injection compared to WT rats and were phenotypically normal as WT rats at 1 year of age [19]. However, these findings contradict those presented in another study of *Bmpr2* mutant rats. Hautefort et al. generated *Bmpr2* mutant rats with a monoallelic deletion of 71 base pairs of exon 1 and showed that these rats developed age-dependent spontaneous PH with penetrance rates as low as 16–27%, similar to the clinical setting [18]. In addition, for the study by Tian et al., the end point for the MCT model was 3 weeks which had similar findings as our study at this time point. However, in the present study, in addition to 3 weeks after MCT treatment, we had several later end points such as 4 and 6 weeks after MCT injection. And the notable significant differences in survival and pulmonary vascular and cardiac structural changes between +/44insG and WT became apparent only at the later endpoints. These differences in findings among the studies may be related to the phenotypic differences in the different types of mutations described above. The worsening of PVD in +/44insG rats is consistent with the findings of a comprehensive review of PAH pathology in patients on PAH specific treatments, which showed that the *Bmpr2* mutation was associated with increased intima-media thickness [39]. The echocardiographic findings (lower cardiac output and TAPSE) in the late stages after MCT injection suggest that the rats plausibly died of heart failure which was evident after 3 weeks of MCT treatment. However, considering the diverse toxicities of MCT, the off-target effects of CRISPR/Cas9 system and the diverse biological effects of BMPR2 [8, 40], we should carefully consider the cause of the increased mortality in genetically mutated animals.

At 3 weeks after MCT injection, PAs with Ki67-immunopositive cells were increased in the lungs of +/44insG rats suggesting increased cell proliferation. Vasoconstriction studies using intrapulmonary PA ring also revealed that the PAs of rats with *Bmpr2* mutation showed impaired PA contraction, at least at some concentrations of PGF2α and ET-1, compared with WT, which was previously reported in MCT-PH in association with PASMC phenotypic changes [41]. The dedifferentiation of PASMC to less-contractile and hyperproliferative phenotype, which has been implicated in both clinical and experimental PH [42–44], was demonstrated in the PAs of +/44insG rats by immunostaining using differentiation state specific markers, SM-2 and SMemb. These phenotypic changes of PASMC in +/44insG rats were consistent with recent studies showing increased PASMC proliferation in induced pluripotent stem cell (iPSC)-derived SMCs carrying a nonsense *Bmpr2* mutation in exon 1 induced by CRISPR/Cas9 [45] and role of the interaction between cGMP and BMP/SMAD signalling in the maintenance of a low proliferative and differentiated PASMC phenotype [46]. These previous observations as well as our findings may support the hypothesis that altered PASMC phenotypes in +/44insG rats may lead to extensive PVD indicated by severe narrowing of the distal PA lumen. Recently BMP9 and BMP10, which serve as high-affinity ligands for activin receptor-like kinase 1(ALK-1) and signal via ALK/BMPR2 complex, was shown to bind directly to PASMC for control of the contractile state [47] and also contribute vascular and cardiac remodelling [48]. However, since the role of BMP9 and BMP10 in pathogenesis of PAH is currently unclear, it may be interesting to see what role the increase or decrease of BMP9 and BMP10 play in progressive PH model in rats with BMPR2 mutation. Extensive research needs to be focused on understanding the phenotypic diversity of SMC in the vascular wall and its relevance to BMPR2 signalling as a determinant [44, 49].

We also demonstrated significant decrease in the number of distal PAs per 100 alveoli. These finding are similar to what was described in an earlier study where conditional *Bmpr2* deletion in pulmonary endothelial cells in mice resulted in increased muscularization and decreased number of distal PAs after hypoxic exposure [37], suggesting the roles of *Bmpr2* in endothelial cell in the phenotypic worsening in the later stages in our model, even though this was unaddressed in this study.

We found that the +/44insG mutant rats had a reduced response to long-term pulmonary vasodilator treatment. Although poor response to PDE type 5 inhibitor in the *Bmpr2* mutation carrier was also shown in clinical studies [33], the mechanism by which *Bmpr2* mutations affect the therapeutic effect is unclear. In the present study, among rats treated with tadalafil for 4 weeks, +/44insG rats had reduced survival associated with higher RV/(LV + S), increased muscularization and fewer distal PAs than WT rats, however, PH and PVD in the tadalafil-treated rats appeared to have improved compared with rats without tadalafil treatment at 4 weeks after MCT injection. In the RV myocardial tissue in the animals surviving at 42 days after MCT injection, we observed extensive perivascular and interstitial fibrosis with greater extent in the +/44insG than WT. Although tadalafil contribute to improved survival by suppressing PVD to some extent, these findings suggest that late deterioration of the clinical phenotype in mutant rats on tadalafil treatment may not be due to only PVD but also to RV dysfunction. Since worse RV hemodynamics in the HPAH than IPAH [50] and more severely affected RV function in mutation carriers than in noncarriers despite a similar afterload [7] were reported in clinical PAH, the findings of RV histology in late-stage of tadalafil-treated +/44insG rats may be relevant to the importance of treatment for RV dysfunction in clinical PAH, especially in the era of PAH specific therapy. Thus, it is important to elucidate the role and molecular mechanism of RV myocardial fibrosis associated with the mutation.

Several limitations should be considered in this study. First, to the best of our knowledge, 43_44insG mutation has not been reported in the patients as a pathogenic mutation for PAH. However, since frame-shift causing single base pair deletion in the exon 1 of *Bmpr2* gene was reported in substantial population of the PAH patients [30, 34], suggesting that the mutation created in this study may be a pathogenic mutation. Second, features of human PAH such as incomplete penetrance or gender dimorphism were not observed in this model. The interspecies differences in genetic background associated with these clinical features and the differences between CRISPR/Cas9-induced genetic mutation and the heredity-linked mutation need to be verified in future studies. In the present study, only male rats were used because of milder PH phenotype in female rats with unclear mechanism. However, since gender difference in survival outcome (higher mortality in male) was shown in patient registries [51, 52], studies using ovariectomized female rats should be conducted to more accurately address gender differences. Third, there is also a methodological limitation. The high mortality rate of the *Bmpr2* mutant rat in late disease stage prevented us from conducting comprehensive assessment with sufficient numbers of animals in order to evaluate hemodynamics, cardiac function, immunohistochemistry and vascular tension measurements to provide further mechanistic insights in this model, even though other parameters such as RV/(LV + S) and morphometry of PAs could be assessed.

In conclusion, the present study demonstrated that a clinically-relevant *Bmpr2* mutation adversely impacts both the natural and post-treatment course of PH in rats with significant effects only in the later stages. These observations well recapitulate the clinical phenotypic differences between mutation carriers and non-carriers and may open up new ways to use this new genetic model for preclinical studies to develop new therapeutic target including pathways associated with phenotypic transition of pulmonary artery smooth muscle cells or right ventricular fibrosis or to elucidate the mutation-type specific role of *Bmpr2* mutation in the pathogenesis and development of PAH.

## Supplementary Information

**Additional file 1: Table S1.** Echocardiographic findings in male rats at 7 weeks of age (baseline). **Table S2.** Basic data of male rats evaluated at 3 weeks after MCT or saline administration. **Table S3.** Basic data of female rats evaluated at 3 weeks after MCT or saline administration. **Table S4.** Basic data of male rats evaluated at 6 months of age. **Table S5.** Basic data of male rats evaluated after exposure to chronic hypoxia for 3 weeks. **Table S6.** Basic data of male rats evaluated at 4 weeks after MCT injection. **Table S7.** Basic data of male rats evaluated at 4 weeks after MCT injection and treated with tadalafil. **Table S8.** Basic data of male rats evaluated at 6 weeks after MCT injection and treated with tadalafil. **Figure S1.** Reduction of phosphorylated AKT (pAKT), a noncanonical downstream substrate proteins of BMPR2 signaling, in rats with Bmpr2 mutation (+/44insG). **Figure S2.** Macrophage infiltration into the lung and the representative images of muscularization of distal pulmonary artery in the monocrotaline-treated rats. **Figure S3.** Collagen deposition and number of vessels in the left and right ventricular myocardium after 3 weeks of monocrotaline injection. **Figure S4.** Pulmonary phenotype in female rats after 3 weeks of monocrotaline injection is similar between wild-type and Bmpr2 mutant rats. **Figure S5.** Pulmonary hemodynamics, right ventricular hypertrophy and histological assessment in male rats at 6 months of age. **Figure S6.** Pulmonary hemodynamics, right ventricular hypertrophy and histological assessment after 3 weeks of chronic hypoxic exposure in male rats. **Figure S7.** Systolic aortic pressure, right ventricular systolic pressure, hematocrit and right ventricular myocardial fibrosis at 4 weeks after monocrotaline injection in wild-type and +/44insG rats. **Figure S8.** Endothelin receptor A levels in lungs before and 3 and 4 weeks after monocrotaline injection. **Figure S9.** Expression of phosphodiesterase type 5 in lungs before, 3 and 4 weeks after monocrotaline injection. **Figure S10.** Tadalafil treatment improves survival of wild-type and Bmpr2 mutant rats 4 weeks after monocrotaline injection. **Figure S11.** Long course treatment with tadalafil from day 14 to day 42 of monocrotaline in male wild-type and +/44insG rats.

## Data Availability

The data underlying this article are available in the article and in its Additional file 1. Further inquiries can be directed to the corresponding author.

## Abbreviations

Ach: Acetylcholine
AOP: Aortic pressure
BMPR2: Bone morphogenetic protein receptor type 2
Cas9: CRISPR-associated protein 9
CRISPR: Clustered regularly interspaced short palindromic repeats
ET-1: Endothelin-1
MCT: Monocrotaline
mPAP: Mean pulmonary artery pressure
%MWT: The percentage of medial wall thickness
PA: Pulmonary artery
PAH: Pulmonary arterial hypertension
PASMC: Pulmonary artery smooth muscle cell
PDE5: Phosphodiesterase type 5
PGF2α: Prostaglandin-F2α
PVD: Pulmonary vascular disease
RV: Right ventricle
RV/(LV + S): Weight ratio of right ventricle/left ventricle plus septum
RVSP: Right ventricular systolic pressure
α-SMA: α-Smooth muscle actin
SM-2: Smooth muscle myosin heavy chain SM-2
SMemb: Nonmuscle myosin heavy chain embryonic isoform
SNP: Sodium nitroprusside
WT: Wild-type
ZFN: Zinc finger nuclease
+/44insG: *Bmpr2* Mutant
