# Supplementary material for: CRISPR-mediated Bmpr2 point mutation exacerbates late pulmonary vasculopathy and reduces survival in rats with experimental pulmonary hypertension
Source: Respir Res. 2022 Apr 8;23:87. doi: 10.1186/s12931-022-02005-w (PMC8994407; doi:10.1186/s12931-022-02005-w)
Supplement: Supplementary file 1 — Additional file 1: Table S1. Echocardiographic findings in male rats at 7 weeks of age (baseline). Table S2. Basic data of male rats evaluated at 3 weeks after MCT or saline administration. Table S3. Basic data of female rats evaluated at 3 weeks after MCT or saline administration. Table S4. Basic data of male rats evaluated at 6 months of age. Table S5. Basic data of male rats evaluated after exposure to chronic hypoxia for 3 weeks. Table S6. Basic data of male rats evaluated at 4 weeks after MCT injection. Table S7. Basic data of male rats evaluated at 4 weeks after MCT injection and treated with tadalafil. Table S8. Basic data of male rats evaluated at 6 weeks after MCT injection and treated with tadalafil. Figure S1. Reduction of phosphorylated AKT (pAKT), a noncanonical downstream substrate proteins of BMPR2 signaling, in rats with Bmpr2 mutation (+/44insG). Figure S2. Macrophage infiltration into the lung and the representative images of muscularization of distal pulmonary artery in the monocrotaline-treated rats. Figure S3. Collagen deposition and number of vessels in the left and right ventricular myocardium after 3 weeks of monocrotaline injection. Figure S4. Pulmonary phenotype in female rats after 3 weeks of monocrotaline injection is similar between wild-type and Bmpr2 mutant rats. Figure S5. Pulmonary hemodynamics, right ventricular hypertrophy and histological assessment in male rats at 6 months of age. Figure S6. Pulmonary hemodynamics, right ventricular hypertrophy and histological assessment after 3 weeks of chronic hypoxic exposure in male rats. Figure S7. Systolic aortic pressure, right ventricular systolic pressure, hematocrit and right ventricular myocardial fibrosis at 4 weeks after monocrotaline injection in wild-type and +/44insG rats. Figure S8. Endothelin receptor A levels in lungs before and 3 and 4 weeks after monocrotaline injection. Figure S9. Expression of phosphodiesterase type 5 in lungs before, 3 and 4 weeks after monocrotaline inje [file 12931_2022_2005_MOESM1_ESM.docx]

**Additional file 1**

**CRISPR-Mediated *Bmpr2* Point Mutation Exacerbates Late Pulmonary Vasculopathy and Reduces Survival in Rats with Experimental Pulmonary Hypertension**

**Kabwe, J. C. et al, *Bmpr2* Mutation Reduces Survival in Rats with PH**

**Contents**

Table S1

Table S2

Table S3

Table S4

Table S5

Table S6

Table S7

Table S8

Figure S1

Figure S2

Figure S3

Figure S4

Figure S5

Figure S6

Figure S7

Figure S8

Figure S9

Figure S10

Figure S11

**Table S1**
**Echocardiographic findings in male rats at 7 weeks of age (baseline)**

|  | Baseline, 7-week old, male | |  |
| --- | --- | --- | --- |
|  | WT | +/44insG | p |
| n | 5 | 5 |  |
| HR (/min) | 351±8 | 343±14 | 0.64 |
| LVDd (mm) | 6.7±0.2 | 6.8±0.1 | 0.56 |
| LVEF (%) | 54.5±3.2 | 52.5±3.0 | 0.67 |
| CO (mL/min) | 113±11 | 106±3 | 0.53 |
| PAAT (msec) | 36±1.5 | 39±1.3 | 0.18 |
| RVWTd (mm) | 0.51±0.01 | 0.45±0.02 | 0.02 |
| TAPSE (mm) | 2.5±0.05 | 2.7±0.07 | 0.06 |

Data are presented as mean ± SEM. WT: wild-type rats, +/44insG: rats with a monoallelic single nucleotide (guanine) insertion in exon 1 of *Bmpr2*, HR: heart rate, LVDd: left ventricular diameter at end-diastole, LVEF: left ventricular ejection fraction, PAAT: pulmonary artery accelerating time, RVWTd: right ventricular wall thickness at end-diastole, TAPSE: the systolic excursion of the tricuspid annular plane. Comparisons between the groups were made using, two-tailed unpaired *t*-test.

**Table S2**

**Basic data of male rats evaluated at 3 weeks after MCT or saline administration**

|  | Control, male | | 3-weeks after MCT, male | |  |
| --- | --- | --- | --- | --- | --- |
|  | WT | +/44insG | WT | +/44insG | P (ANOVA) |
| N | 10 | 12 | 10 | 11 |  |
| Bw (g) | 354±9 | 349±8 | 351±12 | 370±11 | 0.39 |
| HR (/min) | 368±9 | 367±11 | 377±10 | 371±10 | 0.89 |
| mPAP (mmHg) | 15.5±0.9 | 16.4±0.4 | 36.4±3.0*,† | 34.2±2.3*,† | <0.0001 |
| Sys AOP (mmHg) | 144.3±5.3 | 139.8±5.3 | 134.6±7.0 | 127.6±7.4 | 0.27 |
| RV (g) | 0.163±0.005 | 0.127±0.007 | 0.239±0.018*,† | 0.207±0.008*,† | <0.0001 |
| LV+S (g) | 0.557±0.007 | 0.477±0.018* | 0.530±0.019 | 0.510±0.018 | 0.01 |
| Fulton index | 0.29±0.01 | 0.26±0.01 | 0.45±0.03*,† | 0.41±0.01*,† | <0.0001 |

Data are presented as mean ± SEM. Control: rats assessed at 3weeks after saline injection, MCT: monocrotaline, WT: wild-type rats, +/44insG: rats with a monoallelic single nucleotide (guanine) insertion in exon 1 of *Bmpr2*, Bw: body weight, HR: heart rate, mPAP: mean pulmonary artery pressure, Sys AOP: systolic aortic pressure, RV: right ventricle, LV+S: left ventricle + septum. Comparisons between the groups were made using one-way analysis of variance (ANOVA) with Bonferroni's multiple comparisons. *: p<0.05 vs Control, WT,†: p<0.05 vs control, +/44insG

**Table S3**

**Basic data of female rats evaluated at 3 weeks after MCT or saline administration**

|  | Control, female | | 3-weeks after MCT, female | |  |
| --- | --- | --- | --- | --- | --- |
|  | WT | +/44insG | WT | +/44insG | P (ANOVA) |
| n | 5 | 5 | 7 | 6 |  |
| Bw (g) | 229±2 | 235±11 | 239±6 | 238±3 | 0.69 |
| HR (/min) | 389±11 | 375±16 | 360±10 | 373±5 | 0.33 |
| mPAP (mmHg) | 15.0±1.1 | 15.0±0.8 | 26.4±2.5* † | 22.6±1.8 | 0.0005 |
| Sys AOP (mmHg) | 112.8±3.0 | 112.8±3.6 | 138.3±8.7 | 136.2±5.5 | 0.012 |
| RV (g) | 0.114±0.010 | 0.094±0.006 | 0.137±0.009* | 0.124±0.003 | 0.008 |
| LV+S (g) | 0.390±0.022 | 0.340±0.017 | 0.434±0.011* | 0.372±0.015† | 0.003 |
| Fulton index | 0.28±0.01 | 0.28±0.01 | 0.32±0.02 | 0.34±0.01† | 0.019 |

Data are presented as mean ± SEM. Control: rats assessed at 3weeks after saline injection, MCT: monocrotaline, WT: wild-type rats, +/44insG: rats with a monoallelic single nucleotide (guanine) insertion in exon1 of *Bmpr2*, Bw: body weight, HR: heart rate, mPAP: mean pulmonary artery pressure, Sys AOP: systolic aortic pressure, RV: right ventricle, LV+S: left ventricle + septum. Comparisons between the groups were made using one-way analysis of variance (ANOVA) with Bonferroni's multiple comparisons. *: p<0.05 vs Control, WT, †: p<0.05 vs control, +/44insG

**Table S4**

**Basic data of male rats evaluated at 6 months of age**

|  | 6-month old, male | |  |
| --- | --- | --- | --- |
|  | WT | +/44insG | p |
| n | 4 | 11 |  |
| Bw (g) | 497±15 | 476±9 | 0.27 |
| HR (/min) | 377±19 | 377±10 | 0.99 |
| mPAP (mmHg) | 16.0±0.7 | 14.1±0.7 | 0.13 |
| Sys AOP (mmHg) | 136.5±9.7 | 138.5±3.8 | 0.81 |
| RV (g) | 0.193±0.006 | 0.163±0.005 | 0.004 |
| LV+S (g) | 0.782±0.038 | 0.622±0.013 | 0.0002 |
| Fulton index | 0.25±0.02 | 0.26±0.01 | 0.46 |

Data are presented as mean ± SEM. Control: rats assessed at 3weeks after saline injection, MCT: monocrotaline, WT: wild-type rats, +/44insG: rats with a monoallelic single nucleotide (guanine) insertion in exon 1 of *Bmpr2*, Bw: body weight, HR: heart rate, mPAP: mean pulmonary artery pressure, Sys AOP: systolic aortic pressure, RV: right ventricle, LV+S: left ventricle + septum. Comparisons between the groups were made using, two-tailed unpaired *t*-test.

**Table S5**

**Basic data of male rats evaluated after exposure to chronic hypoxia for 3 weeks**

|  | 3-week chronic hypoxia, male | |  |
| --- | --- | --- | --- |
|  | WT | +/44insG | P |
| n | 6 | 5 |  |
| Bw (g) | 369±9 | 351±3 | 0.09 |
| HR (/min) | 353±6 | 372±5 | 0.04 |
| mPAP (mmHg) | 38.5±4.1 | 38.8±1.3 | 0.96 |
| Sys AOP (mmHg) | 144.0±7.5 | 125.5±6.1 | 0.11 |
| RV (g) | 0.266±0.010 | 0.242±0.009 | 0.12 |
| LV+S (g) | 0.552±0.013 | 0.502±0.010* | 0.006 |
| Fulton index | 0.48±0.02 | 0.48±0.02 | 0.99 |

Data are presented as mean ± SEM. Control: rats assessed at 3weeks after saline injection, MCT: monocrotaline, WT: wild-type rats, +/44insG: rats with a monoallelic single nucleotide (guanine) insertion in exon-1 of *Bmpr2*, Bw: body weight, HR: heart rate, mPAP: mean pulmonary artery pressure, Sys AOP: systolic aortic pressure, RV: right ventricle, LV+S: left ventricle + septum. Comparisons between the groups were made using, two-tailed unpaired *t*-test.

**Table S6**

**Basic data of male rats evaluated at 4 weeks after MCT injection**

|  | 4-weeks after MCT, without treatment, male | |  |
| --- | --- | --- | --- |
|  | WT | +/44insG | p |
| n | 12 | 7 |  |
| Bw (g) | 331±12 | 280±11 | 0.002 |
| HR (/min) | 336±12 | 330±14 | 0.75 |
| mPAP (mmHg) | 45.8±3.0 | 55.8±3.8 | 0.07 |
| Sys AOP (mmHg) | 97.3±7.9 | 92.4±9.7 | 0.72 |
| RV (g) | 0.359±0.018 | 0.297±0.005 | 0.02 |
| LV+S (g) | 0.583±0.018 | 0.421±0.006 | <0.0001 |
| Fulton index | 0.62±0.03 | 0.70±0.02 | 0.045 |

Data are presented as mean ± SEM. Control: rats assessed at 3 weeks after saline injection, MCT: monocrotaline, WT: wild-type rats, +/44insG: rats with a monoallelic single nucleotide (guanine) insertion in exon 1 of *Bmpr2*, Bw: body weight, HR: heart rate, mPAP: mean pulmonary artery pressure, Sys AOP: systolic aortic pressure, RV: right ventricle, LV+S: left ventricle + septum. Comparisons between the groups were made using, two-tailed unpaired *t*-test.

**Table S7**

**Basic data of male rats evaluated at 4 weeks after MCT injection and treated with tadalafil**

|  | 4-weeks after MCT, tadalafil treatment, male | |  |
| --- | --- | --- | --- |
|  | WT | +/44insG | p |
| n | 5 | 8 |  |
| Bw (g) | 308±15 | 331±11 | 0.23 |
| HR (/min) | 377±9 | 371±11 | 0.64 |
| mPAP (mmHg) | 40±4.7 | 43.8±4.6 | 0.60 |
| Sys AOP (mmHg) | NA | NA |  |
| RV (g) | 0.306±0.021 | 0.280±0.026 | 0.46 |
| LV+S (g) | 0.579±0.028 | 0.518±0.011 | 0.049 |
| Fulton index | 0.53±0.03 | 0.54±0.05 | 0.86 |

Data are presented as mean ± SEM. Control: rats assessed at 3 weeks after saline injection, MCT: monocrotaline, WT: wild-type rats, +/44insG: rats with a monoallelic single nucleotide (guanine) insertion in exon1 of *Bmpr2*, Bw: body weight, HR: heart rate, mPAP: mean pulmonary artery pressure, Sys AOP: systolic aortic pressure, NA: not available, RV: right ventricle, LV+S: left ventricle + septum. Comparisons between the groups were made using, two-tailed unpaired *t*-test.

**Table S8**

**Basic data of male rats evaluated at 6 weeks after MCT injection and treated with tadalafil**

|  | 6-weeks after MCT, tadalafil treatment, male | |  |
| --- | --- | --- | --- |
|  | WT | +/44insG | p |
| n | 8 | 4 |  |
| Bw (g) | 332±15 | 295±15 | 0.16 |
| HR (/min) | 321±19 | 347±14 | 0.40 |
| mPAP (mmHg) | 33.3±2.8 | 38.7±4.8 | 0.34 |
| Sys AOP (mmHg) | 136.3±5.5 | 130.7±8.8 | 0.58 |
| RV (g) | 0.280±0.018 | 0.339±0.027 | 0.09 |
| LV+S (g) | 0.517±0.015 | 0.508±0.029 | 0.77 |
| Fulton index | 0.54±0.03 | 0.67±0.03 | 0.036 |

Data are presented as mean ± SEM. Control: rats assessed at 3 weeks after saline injection, MCT: monocrotaline, WT: wild-type rats, +/44insG: rats with a monoallelic single nucleotide (guanine) insertion in exon 1 of *Bmpr2*, Bw: body weight, HR: heart rate, mPAP: mean pulmonary artery pressure, Sys AOP: systolic aortic pressure, RV: right ventricle, LV+S: left ventricle + septum. Comparisons between the groups were made using, two-tailed unpaired *t*-test.

**
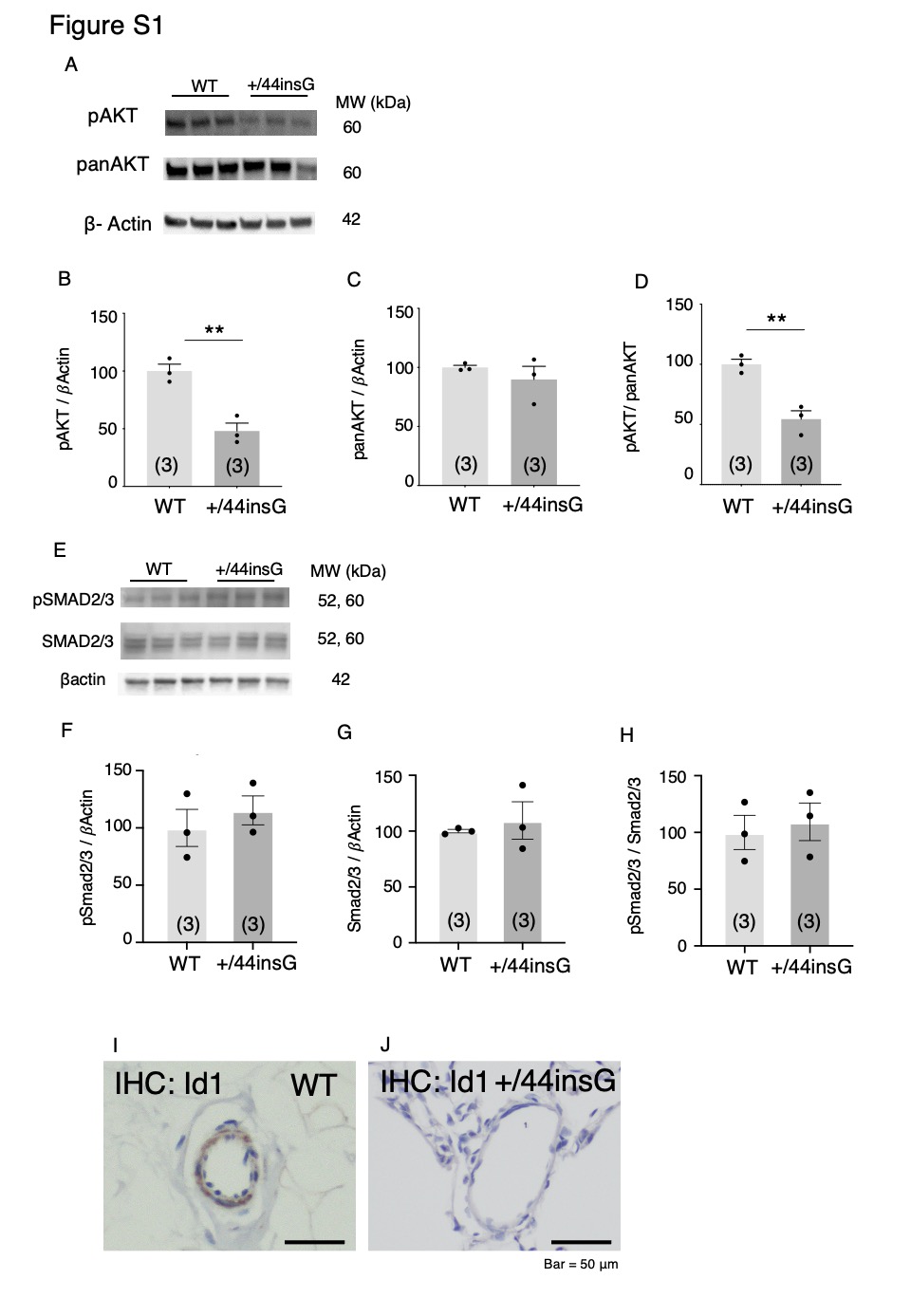
**

**Figure S1**
**Reduction of phosphorylated AKT (pAKT), a noncanonical downstream substrate proteins of BMPR2 signaling, in rats with *Bmpr2* mutation (+/44insG)**
Representative western blots (**A**) and the quantifications of relative protein expression of (**B**) pAKT (n=3 each) and (**C**) total panAKT (n=3 each) in the lung tissue. (**D**) The ratio of pAKT to panAKT in wild-type (WT) and +/44insG rats at 10 weeks of age (n=3 each). **(E)** Representative western blots for pSMAD2/3, SMAD 2/3 and βactin in wild-type (WT) and +/44insG rats (n=3 each).**(F-H)** The quantifications of relative protein expression of (**F**) pSMAD 2/3, (**G**) SMAD 2/3 and (**H**) pSMAD/SMAD 2/3 ratio in WT and +/44insG rats (n=3 each). **(I-J)** Representative immunohistochemistry of Id1 in WT and +/44insG rats respectively. Scale bar: 50μm.

The numbers in parentheses represent the numbers of rats examined. Data are presented as means and SEM; unpaired t-test was used; **p<0.01.

WT=wild-type; +/44insG=rats with bone morphogenetic protein receptor type 2 mutation; pAKT=phosphorylated AKT; MCT= monocrotaline injection

**
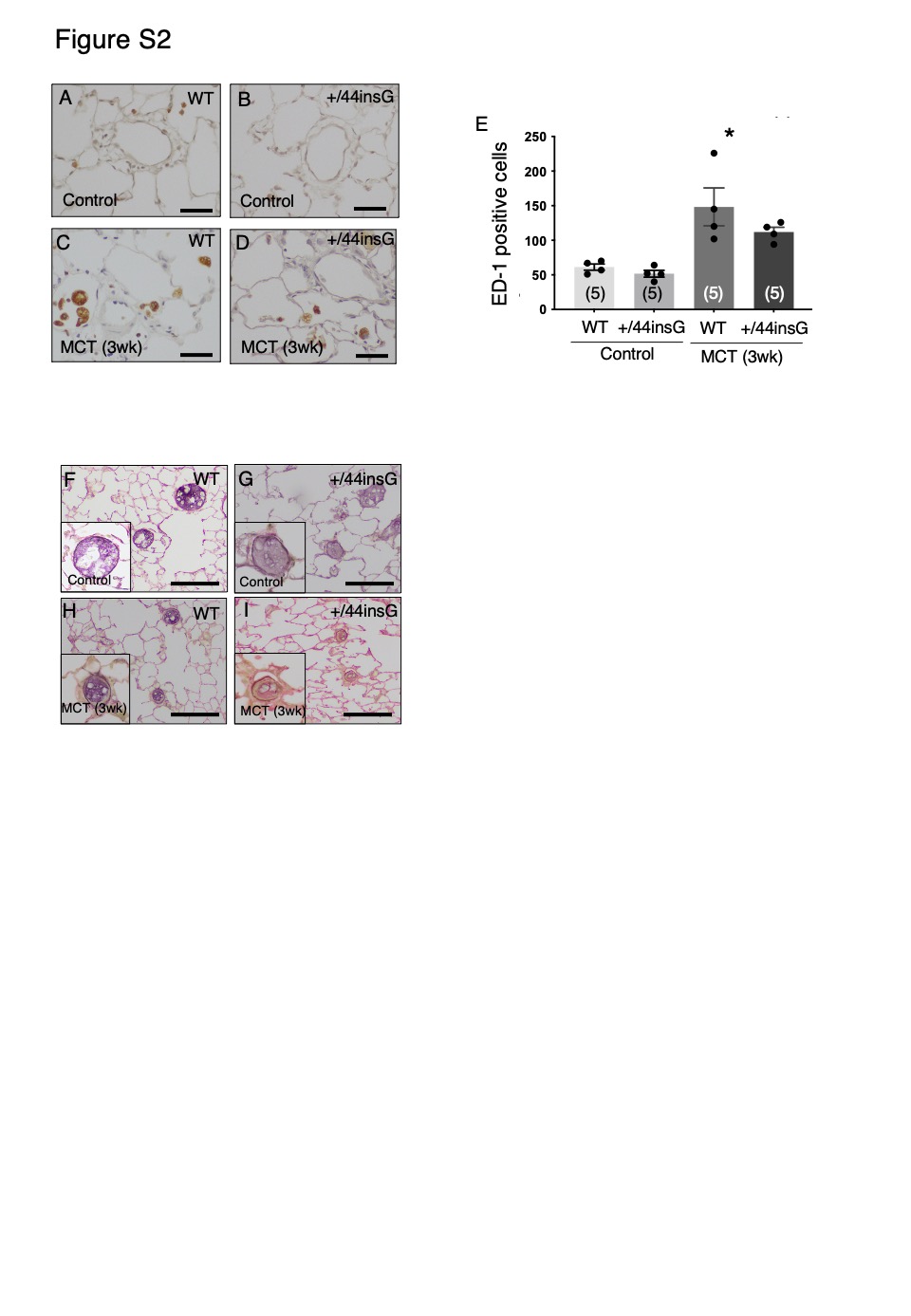
**

**Figure S2**

**Macrophage infiltration into the lung and the representative images of muscularization of distal pulmonary artery in the monocrotaline-treated rats**

(**A**-**D**) Representative images showing ED-1 positive cells in male control and MCT-injected rats (n=20). Scale bar: 25 μm. (**E**) Quantitative analysis of macrophage/monocyte marker ED-1 positive cells. (F-I) Representative images of the distal PAs for the assessment of % muscularization shown in Figure 2D. Scale bar: 200μm. The numbers in parentheses represent the numbers of rats examined. Data are presented as means and SEM; one-way ANOVA followed by Bonferroni’s multiple comparison test.

WT=wild-type; +/44insG=rats with bone morphogenetic protein receptor type 2 mutation; ED-1=macrophage/monocyte marker (ED-1).

**
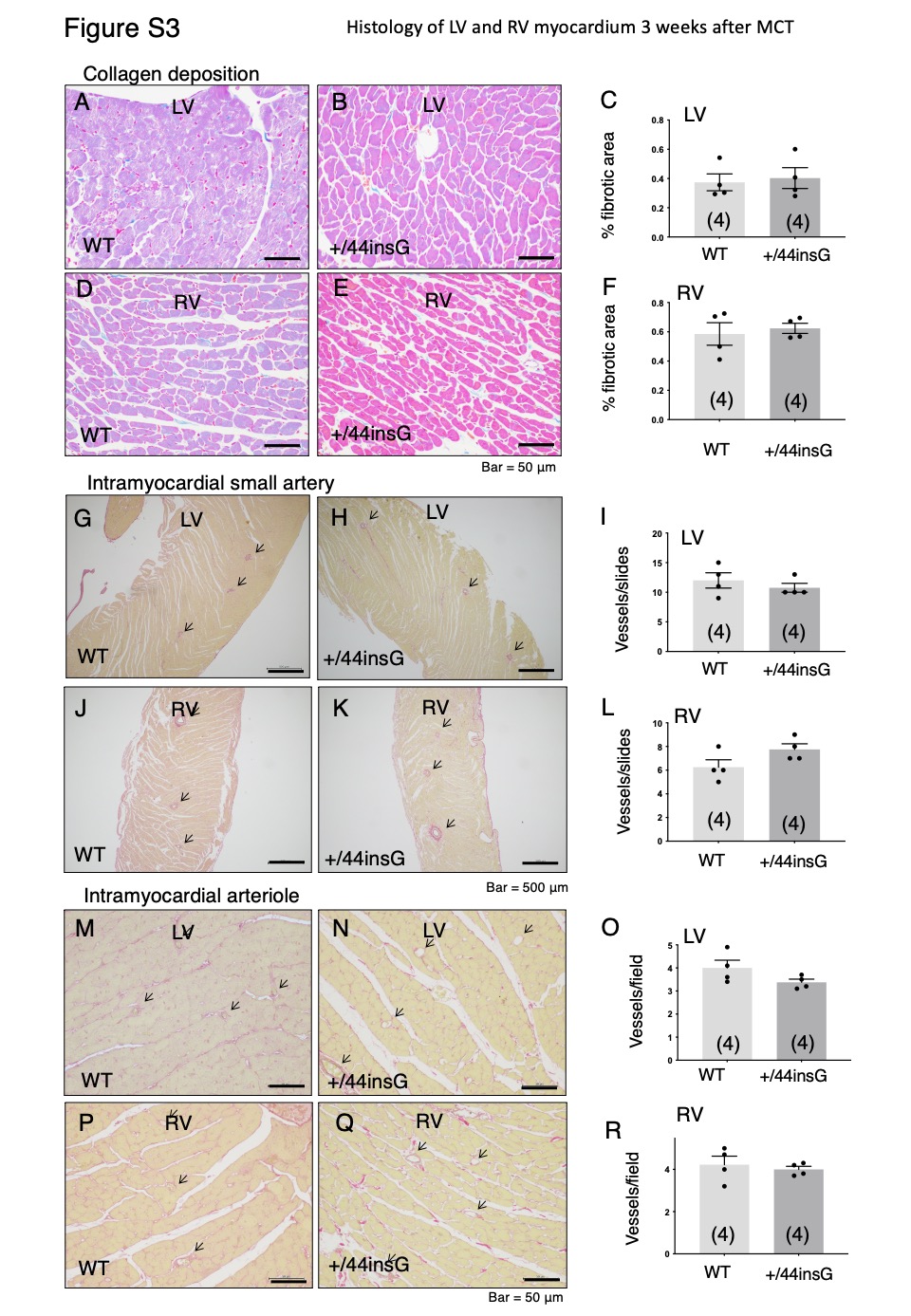
**

**Figure S3
Collagen deposition and number of vessels in the left and right ventricular myocardium after 3 weeks of monocrotaline injection**(**A**, **B**, **D** and **E**) Representative images of myocardial tissue from left ventricle (LV) and right ventricle (RV) by Masson's trichrome stain in rats 3 weeks after MCT injection in both WT and +/44insG rats and (**C** and **F**) the quantification of myocardial fibrosis (n=4 for each group). Scale bar: 50μm. (**G**, **H**, **J**,**K**, **M**, **N**, **P** and **Q**) Representative images of myocardial tissue from left ventricle (LV) and right ventricle (RV) stained by Elastica van Gieson staining in rats 3 weeks after MCT injection in both WT and +/44insG rats and (**I** and **L**) the quantification of the number of intramyocardial small arteries (vessels in diameter 50-250 μm, n=4 for each group). Scale bar: 500μm. (**O** and **R**) The quantification of the number of intramyocardial arterioles (vessels in diameter 10-50 μm, n=4 for each group). Scale bar: 50μm. The numbers in parentheses represent the numbers of male rats examined. Data are presented as means and SEM; unpaired t-test was used.

WT=wild-type; +/44insG=rats with bone morphogenetic protein receptor type 2 mutation; MCT=monocrotaline; LV= left ventricle, RV=right ventricle.

**
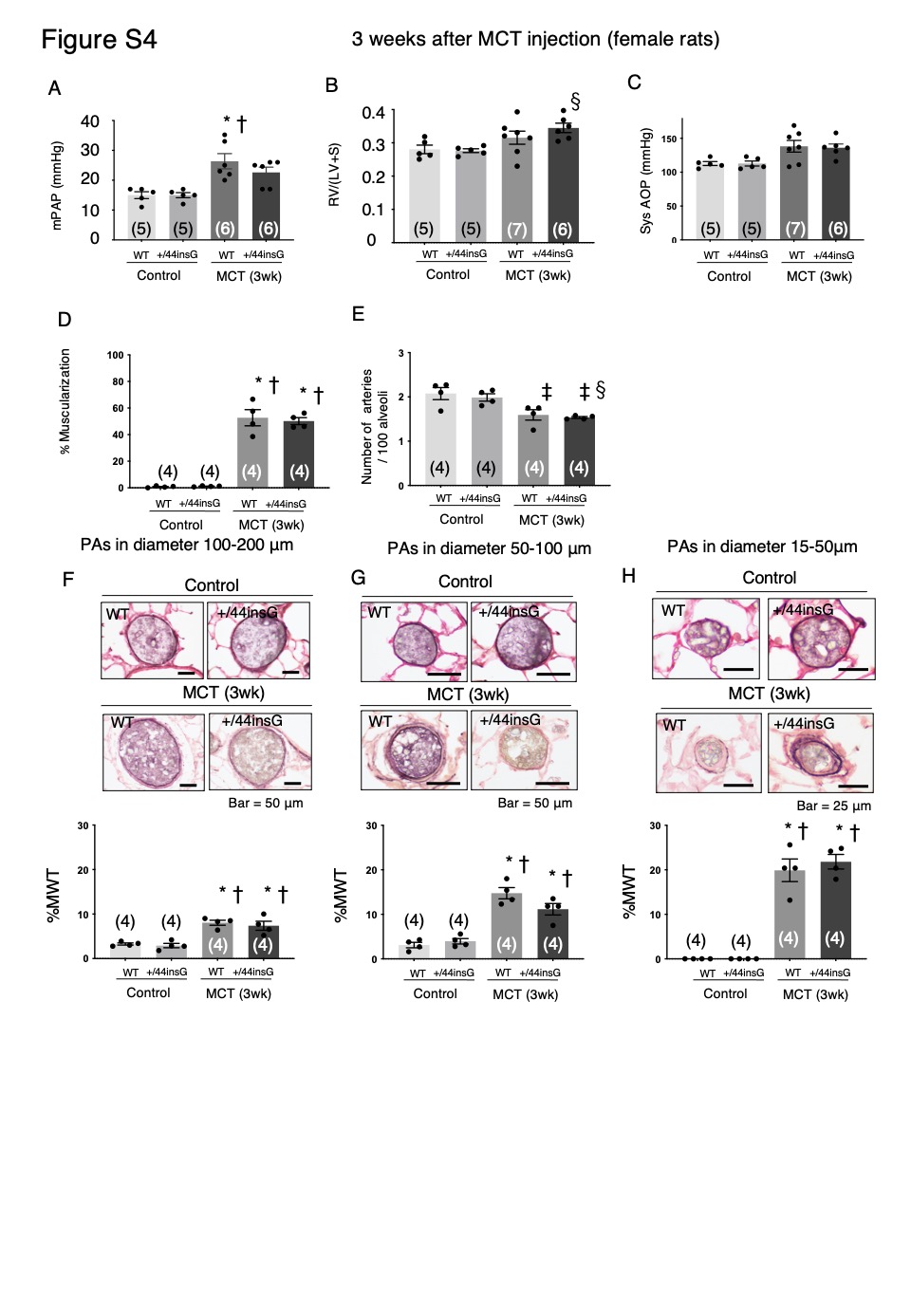
**

**Figure S4
Pulmonary phenotype in female rats after 3 weeks of monocrotaline injection** **is similar between wild-type and *Bmpr2* mutant rats**All the measurements were done in female wild-type (WT) and *Bmpr2* mutation (+/44insG) in control rats and monocrotaline (MCT) treated rats at 3 weeks after injection. **(A)** Assessment of mean pulmonary artery pressure (mPAP) (n=22, WT and +/44insG),**(B)**the weight ratio of right ventricle/left ventricle plus septum (RV/[LV+S]) (n=23 WT and +/44insG),**(C)** systolic aortic pressure (Sys AOP) measured in control rats and MCTtreated rats (n=23 WT and +/44insG). **(D)** The quantification of the muscularisation of distal PAs (n= 16, WT and +/44insG) and **(E)** the quantification of the distal PAs per 100 alveoli (n=16, WT and +/44insG) in control and MCTtreated rats. (**F-H)** Representative images of barium filled PAs with diameter 100-200μm (**F**),50-100μm (**G**), 15-50μm (**H**) in control (top images) and MCT-treated rats at 3 weeks after injection (bottom images) and the quantification of medial wall thickness (% MWT)(n=16, WT and +/44insG). Scale bar: 50μm (**F** and **G**), 25μm (**H**).

The numbers in parentheses represent the numbers of rats examined. Data are presented as means ± SEM; one-way ANOVA followed by Bonferroni’s multiple comparison test was used;*：P <0.01 vs Control, WT; †: P <0.01 vs Control, +/44insG; ‡: P <0.05 vs Control, WT;§:P <0.05 vs Control, +/44insG

mPAP=mean pulmonary artery pressure; RV/(LV+S) =weight ratio of right ventricle/left ventricle plus septum; Sys AOP = systolic aortic pressure; WT=wild-type; +/44insG=rats with bone morphogenetic protein receptor type 2 mutation; MCT= monocrotaline; PAs = pulmonary arteries; %MWT = percentage of medial wall thickness; wk= weeks.

**
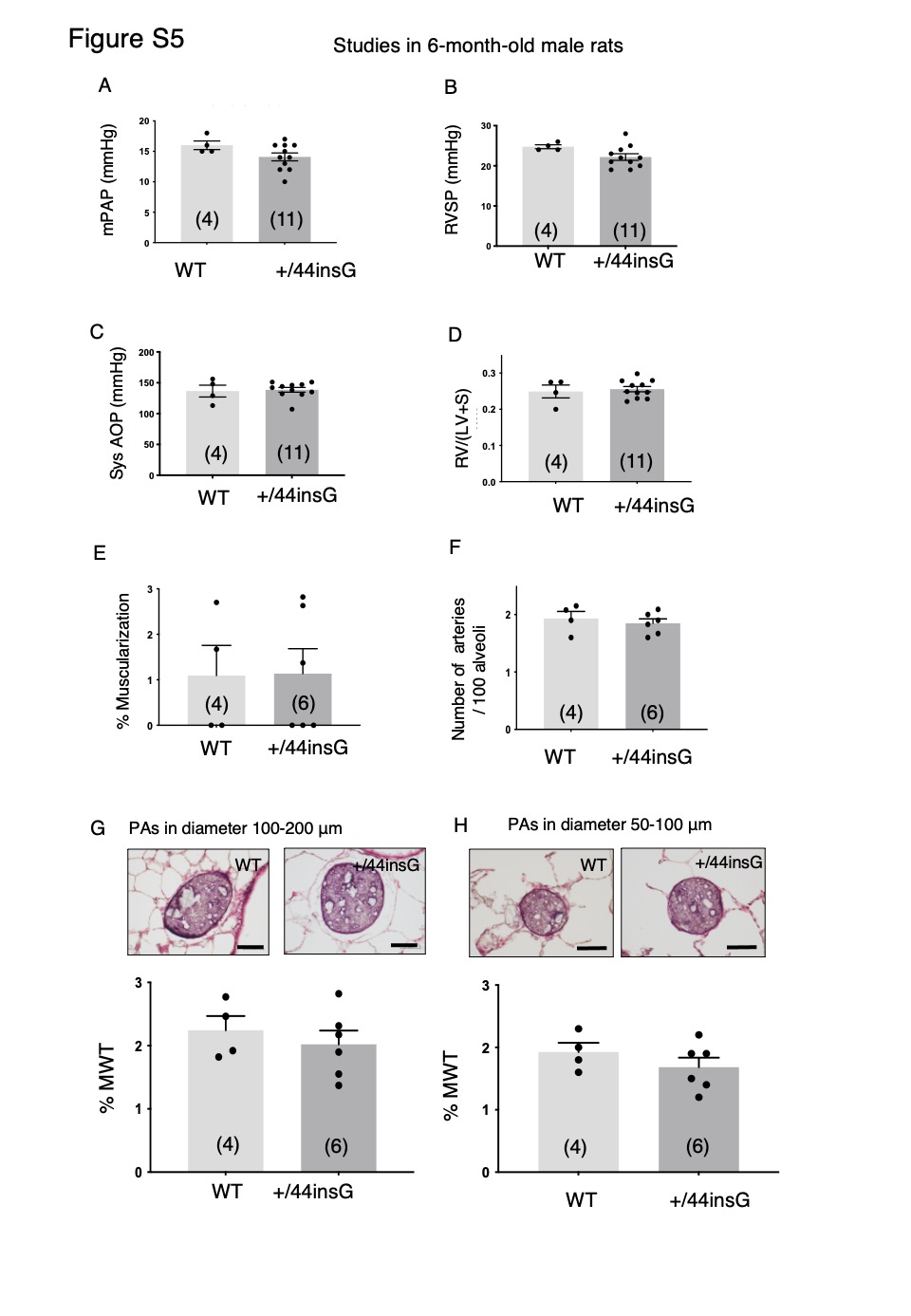
**

**Figure S5
Pulmonary hemodynamics, right ventricular hypertrophy and histological assessment in male rats at 6 months of age**
+/44insG rats were compared to the wild-type (WT) littermate. (**A**) Assessment of mean pulmonary artery pressure (mPAP) (n=15, WT and +/44insG), (**B**) right ventricular systolic pressure (RVSP) (n=15, WT and +/44insG), (**C**) systolic aortic pressure (SysAOP)(n=15, WT and +/44insG). (**D**) weight ratio of right ventricle/left ventricle plus septum (RV/[LV+S]) (n=15,WT and +/44insG) respectively. **(E)** Percent (%) muscularisation of distal PAs (15-50μm, n=10,WT and +/44insG). **(F)** Number of arteries per 100 alveoli (n=10, WT and +/44insG). (**G** and **H**) Representative images of barium filled distal pulmonary arteries (PAs) in diameter 100-200μm and 50-100μm with analysis for percent of medial wall thickness (% MWT) (n=10, WT and +/44insG). Scale bar: 50μm. The numbers in parentheses represent the numbers of rats examined. Data are presented as means and SEM; unpaired t- test was used.

mPAP=mean pulmonary artery pressure; WT=wild-type; +/44insG=rats with bone morphogenetic protein receptor type 2 mutation; RVSP=right ventricular systolic pressure; RV/(LV+S) =weight ratio of right ventricle/left ventricle plus septum; Sys AOP=systolic aortic pressure; PAs=pulmonary arteries; %MWT=percentage of medial wall thickness

**
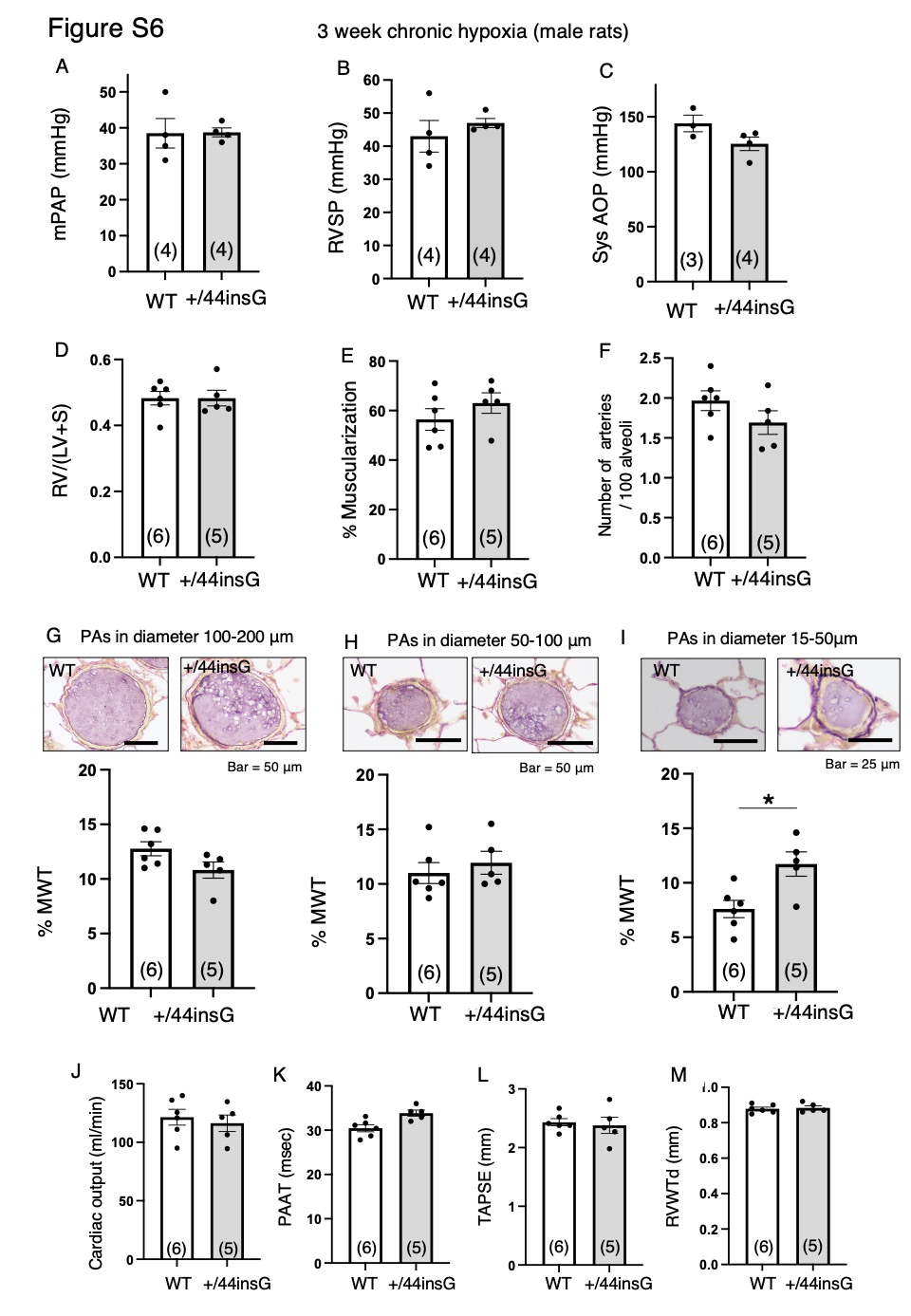
**

**Figure S6
Pulmonary hemodynamics, right ventricular hypertrophy and histological assessment after 3 weeks of chronic hypoxic exposure in male rats**

All the measurements were done after exposure to chronic hypobaric hypoxia for 3 weeks in male wild-type (WT) and *Bmpr2* mutation (+/44insG) rats. **(A)** Assessment of mean pulmonary artery pressure (mPAP) (n=8, WT and +/44insG), (**B**) right ventricular systolic pressure (n=8, WT and +/44insG)**, (C)** systolic aortic pressure (Sys AOP) (n=7, WT and +/44insG) and **(D)** the weight ratio of right ventricle/left ventricle plus septum (RV/[LV+S]) (n=11 WT and +/44insG). **(E)** The quantification of muscularisation of distal PAs (n= 11, WT and +/44insG). **(F)** The quantification of the distal PAs per 100 alveoli (n=11, WT and +/44insG). **(G**-**I)** Representative images of barium filled PAs with diameter 100-200μm(**G**), 50-100μm(**H**) and 15-50μm (**I**) both WT and +/44insG with the quantification of medial wall thickness (%MWT) (n= 11, WT and +/44insG). Scale bar: 50 μm (**G** and **H**), 25μm (**I**). **(J)** Measurement of cardiac output, **(K)** pulmonary artery acceleration time (PAAT), **(L)** tricuspid annular plane systolic excursion (TAPSE), and **(M)** right ventricular wall thickness in diastole (RVWTd) in WT and +/44insG rats after 3 weeks hypoxic exposure (n=11, WT and +/44insG).

The numbers in parentheses represent the numbers of rats examined. Data are presented as means ± SEM; unpaired t- test was used.

mPAP=mean pulmonary artery pressure; RVSP= right ventricular systolic pressure, Sys AOP= systolic aortic pressure, RV/(LV+S) =weight ratio of right ventricle/left ventricle plus septum; WT=wild-type; +/44insG=rats with bone morphogenetic protein receptor type 2 mutation; PAs = Pulmonary arteries; %MWT = percentage of medial wall tension; PAAT=pulmonary artery acceleration time; TAPSE: tricuspid annular plane systolic excursion; RVWTd: right ventricular wall thickness in diastole.


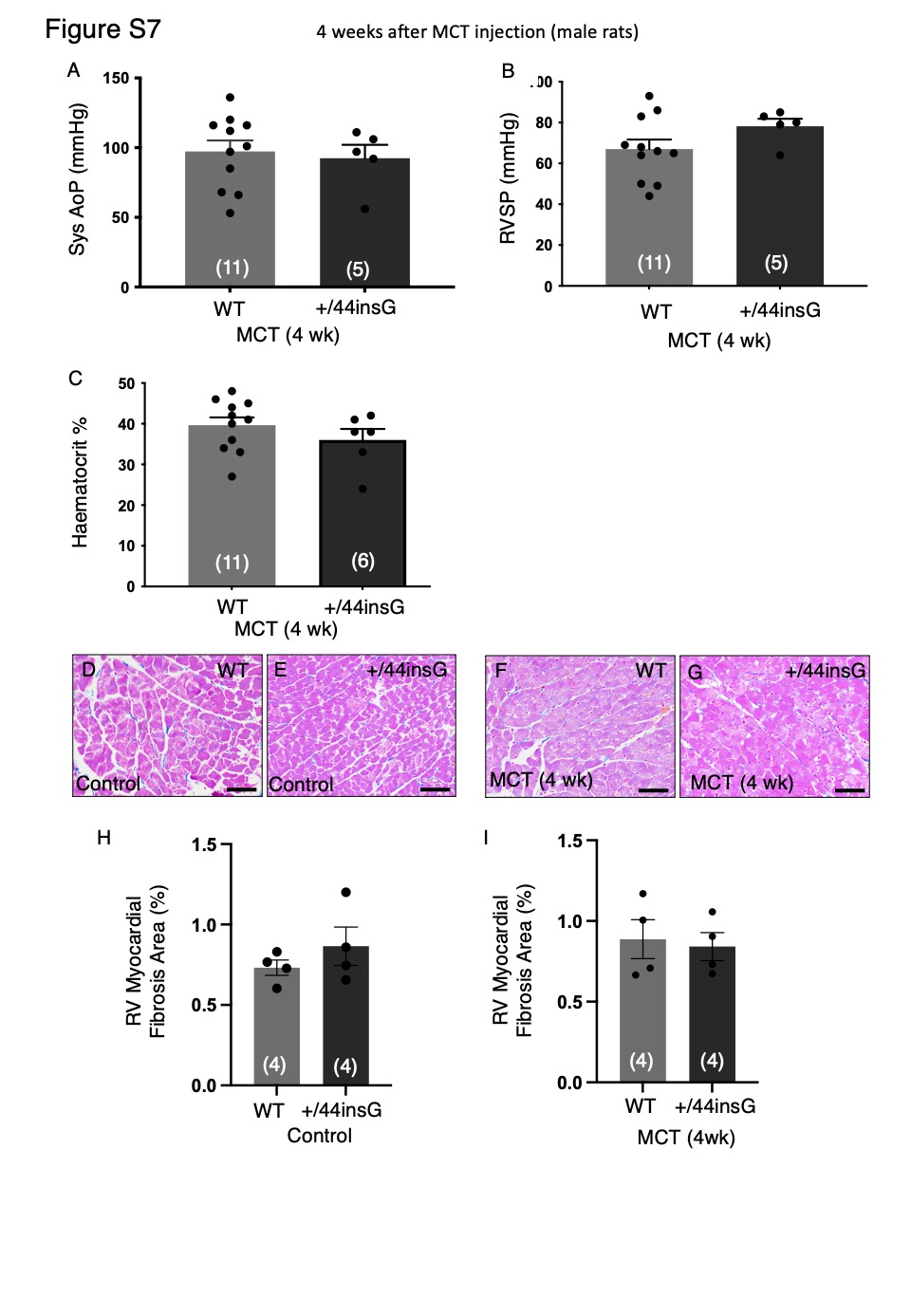


**Figure S7
Systolic aortic pressure, right ventricular systolic pressure, hematocrit and right ventricular myocardial fibrosis at 4 weeks after monocrotaline injection in wild-type and +/44insG rats**
(**A**) Analysis of systolic aortic pressure (Sys AOP, n=16WT and +/44insG), (**B**) right ventricular systolic pressure (RVSP) (n=16,WT and +/44insG) and **(C)** hematocrit (n=17 WT and +/44insG) at 4 weeks after monocrotaline (MCT) injection in wild-type (WT) and +/44insG rats. (**D-G**) Representative images of right ventricle (RV) myocardial tissue by Masson's trichrome stain in control and day 28 MCT in both WT and +/44insG rats and (**H**) the quantification of RV myocardial fibrosis in WT and +/44insG in control and 4 weeks after MCT injection (n=4 for each group). Scale bar: 50μm. The numbers in parentheses represent the numbers of rats examined. Data are presented as means and SEM; unpaired t-test was used.

Sys AOP=systolic aortic pressure; WT=wild-type; +/44insG=rats with bone morphogenetic protein receptor type 2 mutation; MCT=monocrotaline; RVSP=right ventricular systolic pressure; RV=right ventricle.

**
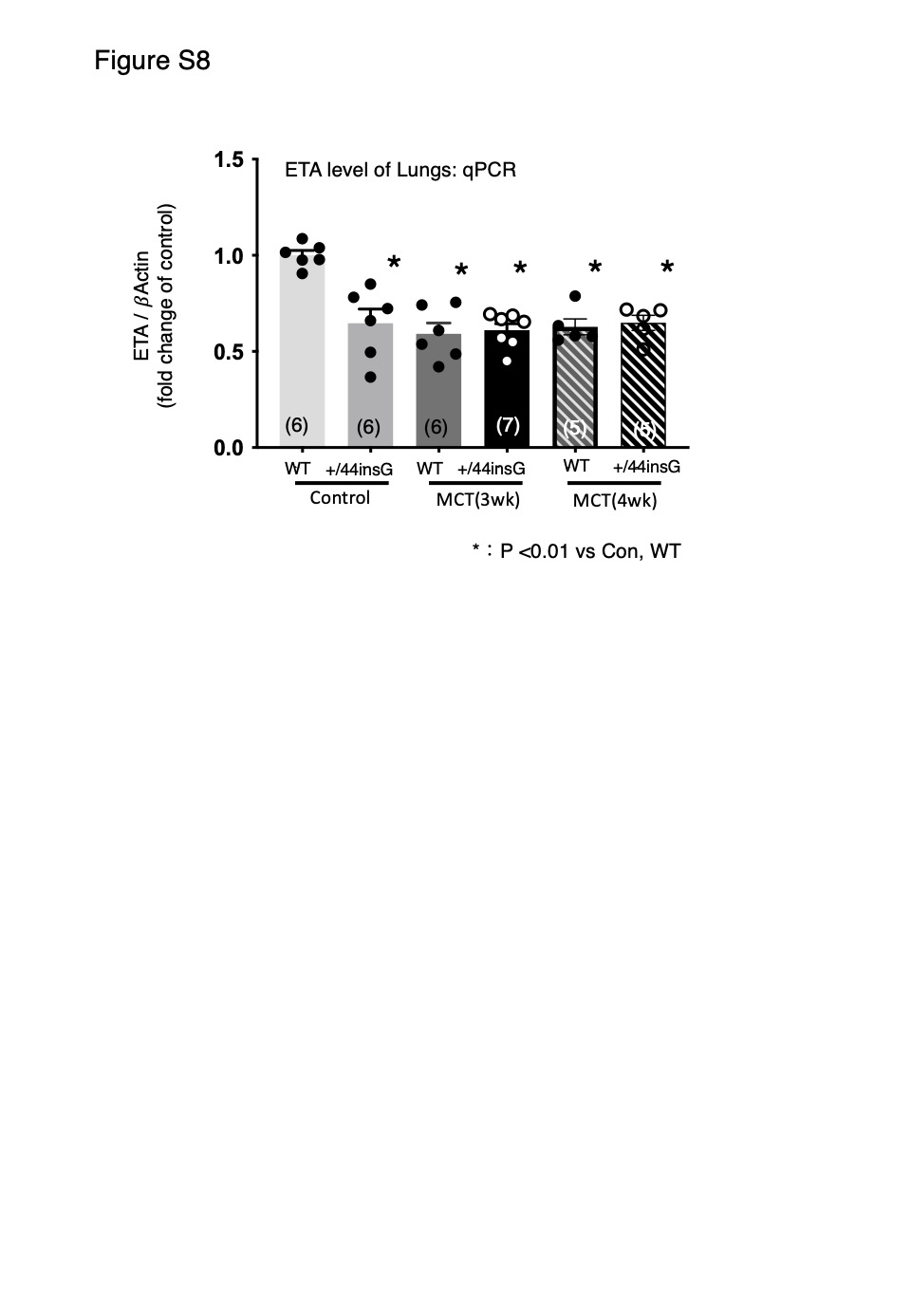
**

**Figure S8**

**Endothelin receptor A levels in lungs before, 3 and 4 weeks after monocrotaline injection.**The expression of endothelin receptor A mRNA normalized to beta actin determined by quantitative real-time PCR in the lungs from control rats (3 weeks after saline injection) and rats 3 weeks and 4 weeks after MCT injection.
The numbers in parentheses represent the numbers of rats examined. Values are presented as the mean ± SEM. One-way analysis of variance (ANOVA) followed by Bonferroni’s multiple comparisons test was used for the analysis. *: P <0.01 vs Control, WT.

**
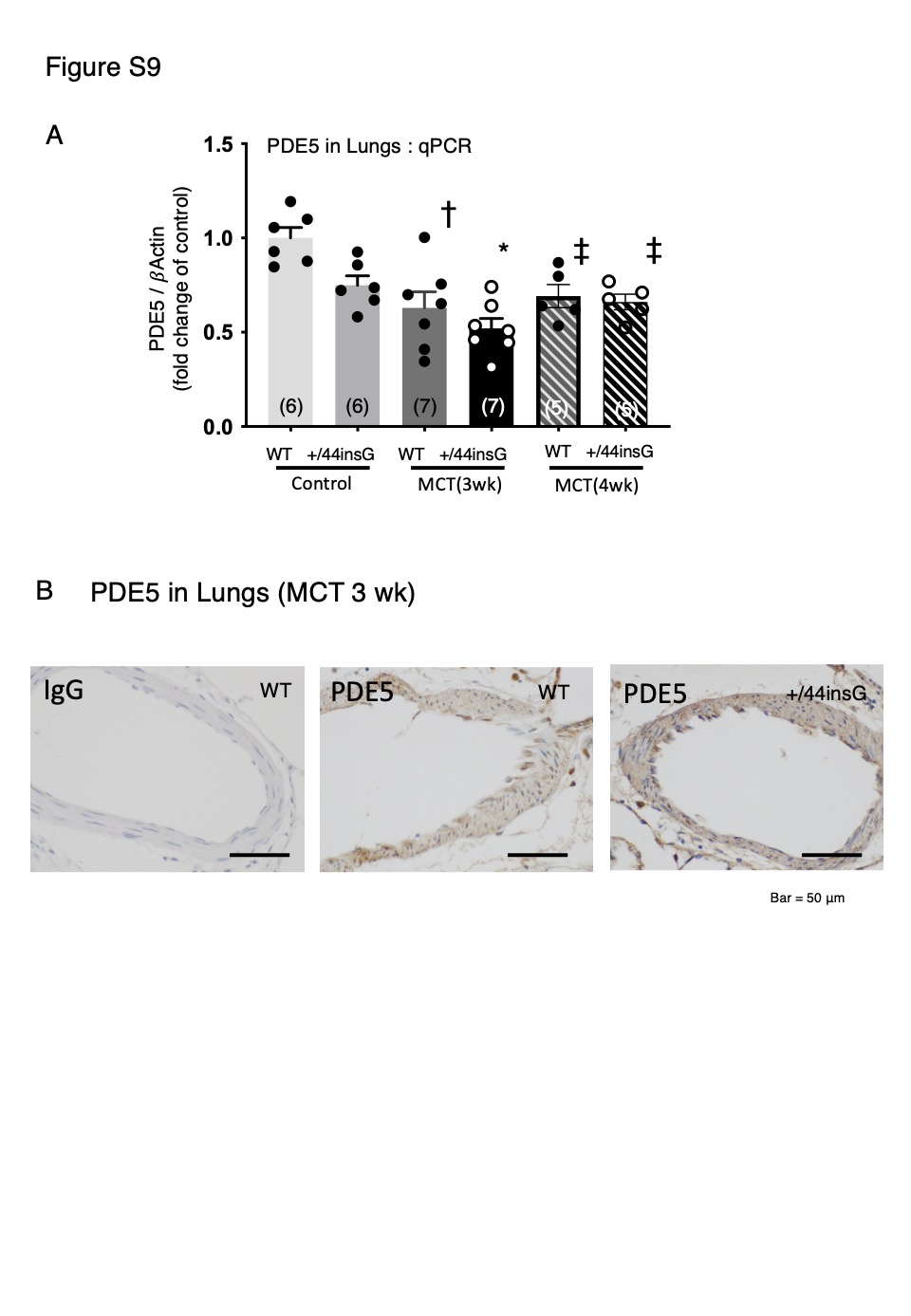
**

**Figure S9
Expression of phosphodiesterase type 5 in lungs before, 3 and 4 weeks after monocrotaline injection**(**A**) The expression of phosphodiesterase type5 mRNA normalized to beta actin determined by quantitative real-time PCRin the lungs from male control rats (3 weeks after saline injection) and male rats 3 weeks and 4 weeks after MCTinjection. (**B**) Immunohistochemical staining of the lungs from wild-type and +/44insG rats at 3weeks after MCTinjection using anti-PDE5 antibody. Scale bar: 50μm. The numbers in parentheses represent the numbers of rats examined. Values are presented as the mean ± SEM. One-way analysis of variance (ANOVA) followed by Bonferroni’s multiple comparisons test was used for the analysis. *: P <0.0001 vs Control, WT; †: P <0.01 vs Control, WT; ‡: P <0.05 vs Control, WT.

**
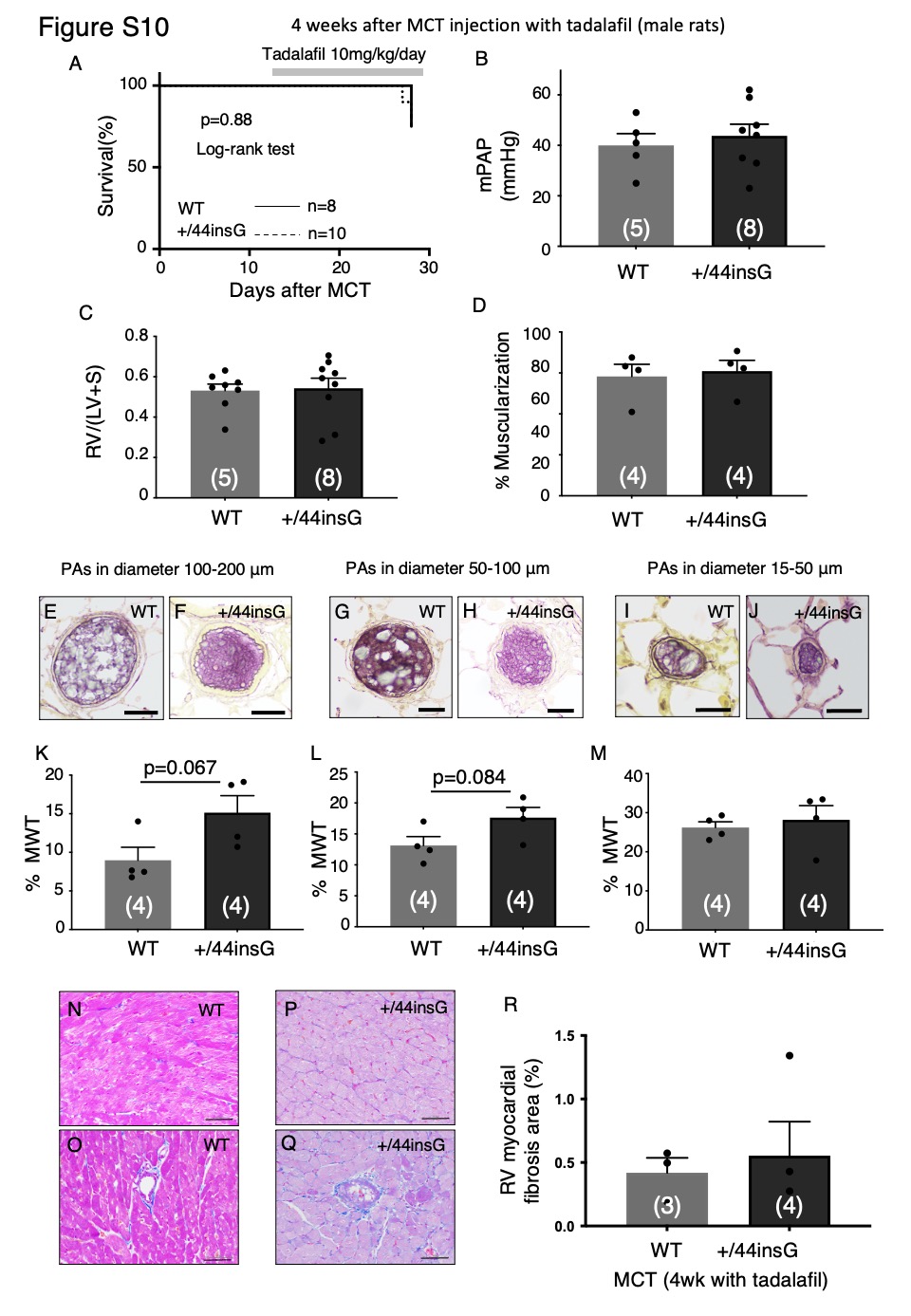
**

**Figure S10
Tadalafil treatment improves survival of wild-type and *Bmpr2* mutant rats 4 weeks after monocrotaline injection**Survival study of male rats with monocrotaline (MCT) induced pulmonary hypertension (PH) that were administered tadalafil 10mg/kg body weight from day 14 to 28 after MCT injection (n=18). (**A**) Survival outcomes of wild-type (WT) and *Bmpr2* mutant (+/44insG) rats. (**B**) Assessment for the mean pulmonary artery pressure (mPAP) of rats surviving on day 28 after MCT (n=13, WT and +/44insG rats). (**C**) The weight ratio of right ventricle/left ventricle plus septum (RV/[LV+S]) (n=13, WT and +/44insG rats). (**D**)The quantification of percent (%) muscularization of distal pulmonary arteries (PAs) (n=8, WT and +/44insG rats). (**E**-**J**) Representative images depict the % medial wall thickness (%MWT) of barium filled vessels sized 100-200, 50-100 and 15-50 μm in WT and +/44insG rats respectively. Scale bar: 50, 25 and 10 μm respectively. (**K**-**M**)The quantification of the MWT % in vessels sized 100-200 μm, 50-100 μm and 15-50 μm in WT and +/44insG rats respectively (n=8,WT and +/44insG rats).(**N-Q**) Representative images of right ventricular myocardial tissue by Masson's trichrome stain and (**R**) the quantification of right ventricular myocardial fibrosis in WT and +/44insG rats with tadalafil treatment at 4 weeks after MCT injection (n= 7, WT and +/44insG rats). Scale bar: 50μm. The numbers in parentheses represent the numbers of rats examined.
For panel **A**, data are presented by Kaplan- Meier survival plot; for panel **B-D**, the data are presented as means and SEM; Log-rank (Mantel-Cox) test for panel **A** and unpaired t-test for panels **B-D** were used**.** p values are indicated in the graph.

WT=wild-type; +/44insG=rats with bone morphogenetic protein receptor type 2 mutation; MCT=monocrotaline; mPAP=mean pulmonary artery pressure; RV/(LV+S)=weight ratio of right ventricle/left ventricle plus septum; PAs=pulmonary arteries; %MWT=the percentage of medial wall thickness.

**
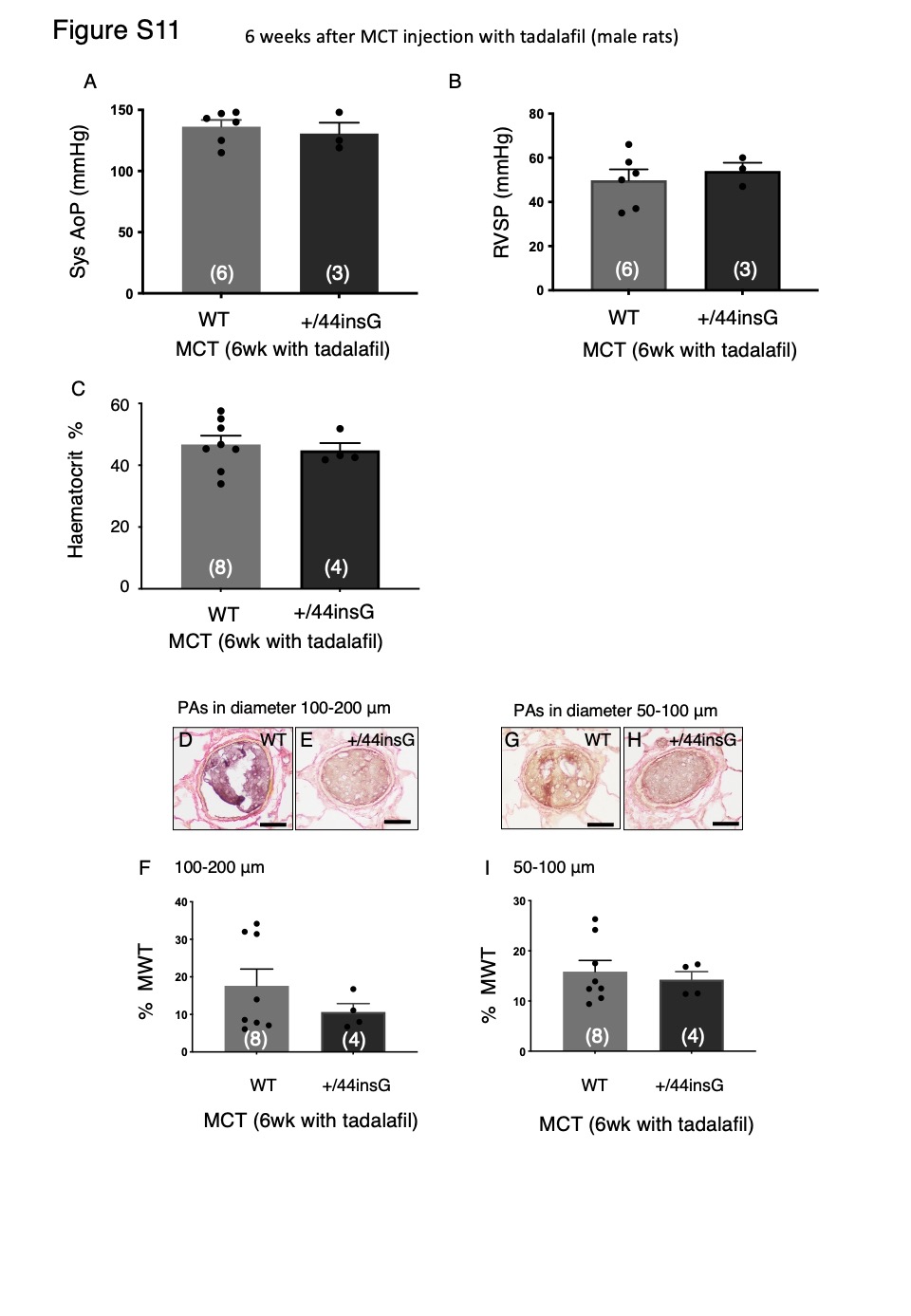
**

**Figure S11
Long course treatment with tadalafil from day 14 to day 42 of monocrotaline in male wild-type and +/44insG rats**
(**A**) Systolic aortic pressure (sys AOP) (n=9, WT and +/44insG rats), (**B**) right ventricle systolic pressure (RVSP) (n=9, WT and +/44insG rats) and (**C**) haematocrit (n=12, WT and +/44insG rats). Data are presented as means and SEM; unpaired t-test was used to statistically analyse the data. (**D-H**) Representative images for the percentage of medial wall thickness (%MWT) in WT and *Bmpr2* mutant (+/44insG) rats (n=12, WT and +/44insG rats) in barium filled vessels in diameters 100-200 and 50-100 μm. Scale bar: 50 μm and 25 μm. (**F** and **I**) The quantification of the %MWT in WT and +/44insG rats in vessels sized 100-200 and 50-100 μm respectively. The numbers in parentheses represent the numbers of rats examined. Unpaired t-test was used**.**

Sys AOP=systolic aortic pressure; WT=wild-type; +/44insG=rats with bone morphogenetic protein receptor type 2 mutation; MCT=monocrotaline; RVSP=right ventricular systolic pressure; PAs=pulmonary arteries; %MWT=the percentage of medial wall thickness.
